# Preserving visual acuity: a compelling 12-year case study of controlling neovascular age-related macular degeneration

**DOI:** 10.1186/s12886-024-03387-9

**Published:** 2024-03-18

**Authors:** Rita O. Tomás, António Campos, Nuno Oliveira, Pedro Soares, João P. Sousa

**Affiliations:** 1 Ophtalmology Department, Centro Hospitalar de Leiria, R. de Santo André, Leiria, 2410-197 Portugal; 2 ciTechCare, Center for Innovative Care and Health Technology, Polytechnic Institute of Leiria, Leiria, Portugal; 3 Software Engineering Department, Doing Software Right Europe (DSRE), Porto, Portugal; 4 Sciences Department, Faculty of Health Sciences, University of Beira Interior, Covilhã, Portugal

**Keywords:** Neovascular age-related macular degeneration, Visual acuity, Pro re nata, Treat and extend, Number of injections, Real-world

## Abstract

**Introduction:**

In neovascular age-related macular degeneration (nAMD) trials, anti-VEGF injection frequency decreases after the first year, while outcomes remain primarily related to the number of injections. To the best of our knowledge, there are no reports of maintaining the best corrected visual acuity (BCVA) for more than 7 years in extension studies.

**Objective:**

To report a 12-year follow-up of a real-world case of nAMD where BCVA was preserved from declining.

**Case description:**

A 67-year-old Caucasian female presented to our department in June 2010 due to decreased vision in her left eye (LE) within the preceding months. Examination showed a BCVA of 85 letters (L) in the right eye (RE) and 35 L in the LE. Fundus examination showed drusen in the macula of both eyes. Macular edema, loss of the macular lutein pigment, macular hypo/hyperpigmentation were observed in the LE. A diagnosis of Type 2 choroidal neovascular membrane (CNV) in the LE was established and within two months a Type 1 CNV developed in the RE. She undergone 9 injections of bevacizumab (six) and ranibizumab (three) within the first year of treatment in the LE and seven injections of ranibizumab within the first year in the RE.

**Results:**

The LE had a mean of 5.2 injections per year, and the RE had a mean of 7.5 injections per year, from 2010 to 2022. RE's BCVA dropped by 8L (85L to 77L) and central retinal thickness (CRT) increased by 16 μm (276 μm to 292 μm) while LE’s BCVA increased by 28L (35L to 63L) and CRT decreased by 369 μm (680 μm to 311 μm), at the twelfth year.

**Conclusions:**

Although the final visual outcome depends on baseline BCVA and lesion type or size, the number of injections is paramount in preserving BCVA and achieving favorable functional outcomes in nAMD, even after 12 years of treatment.

**Supplementary Information:**

The online version contains supplementary material available at 10.1186/s12886-024-03387-9.

## Introduction

Age related macular degeneration (AMD) is the most common cause of blindness after the 7th decade of life in developed countries [1]. Its advanced forms are characterized by choroidal neovascular membrane (CNV) or geographic atrophy. Anti-VEGF agents are the mainstay of the treatment of neovascular AMD (nAMD) [2].

The outcomes are primarily related with the number of injections and not necessarily with the treatment regimen, while the number of injections per year is expected to decrease after the 1st year [3–6]. Unfortunately, the best corrected visual acuity (BCVA) of eyes under treatment declines over time [7]. This fact has been associated with under-treatment and with the development of atrophy [8].

Therefore, the objective of this case report is to present a 12-year follow-up of a real-world case of nAMD where BCVA was maintained with a proactive approach.

## Methods

The patient provided written informed consent to be inserted in the online platform Retina.pt of the Portuguese Retina Group (GER). The study was approved by the Ethical Committee of the Leiria Hospital Center and informed consent was obtained from the patient. Data were retrospectively collected from Retina.pt and from institutional clinical files. Optical coherence tomography (OCT) was performed by certified site personnel using spectral domain OCT (SD-OCT) Spectralis, Heidelberg GmbH, Germany. Angiograms were obtained using the same device’s angiograph. Images were taken using the high resolution (HR) mode with a signal strength of at least 20. CRT values were automatically taken from the center of the fovea and follow-up comparisons were automatically performed by the device’s software. BCVA was obtained using certified Early Treatment Diabetic Retinopathy Study Group (ETDRS) logarithmic charts as it is current practice in the Retina Unit of our Department.

## Case presentation

### Patient information

**Fig. 1:**
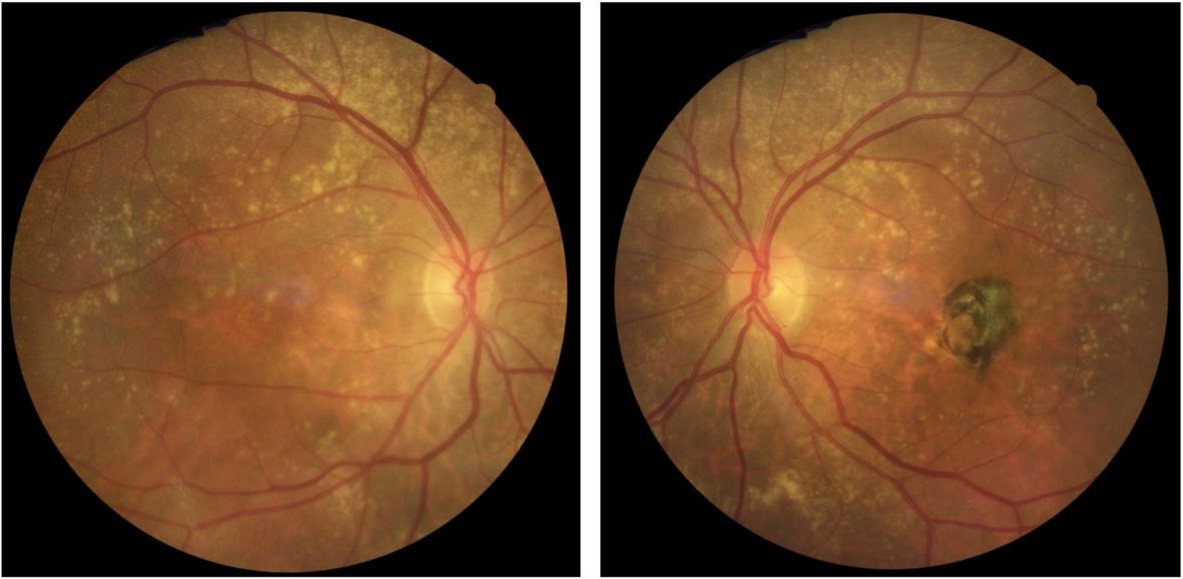
Colour fundus photography (CFP). RE on the left and LE on the right. The RE’s CPF shows macular and extramacular drusen and the loss of coloration inferior and nasal to the fovea suggests edema. In the LE’s, the association of a central discoloration surrounded by a ring of hyperpigmentation suggests atrophy and a RPE tear

A 67-year-old Caucasian female presented to our department in June 2010 due to decreased vision in her left eye (LE) within the preceding months. BCVA was 35 letters (L). The right eye (RE)’s BCVA was 85L. Anterior segment examination and ocular tension were normal. Fundus examination showed drusen in the posterior pole of both eyes, macular edema in the LE at presentation, and developing within two months in the RE as well, some loss of the lutein pigment of both eyes and macular hypo/hyper pigment changes and atrophy in the LE that were attributed to a retinal pigment epithelium (RPE) tear (Fig. 1).

### Diagnostic assessment

**Fig. 2:**
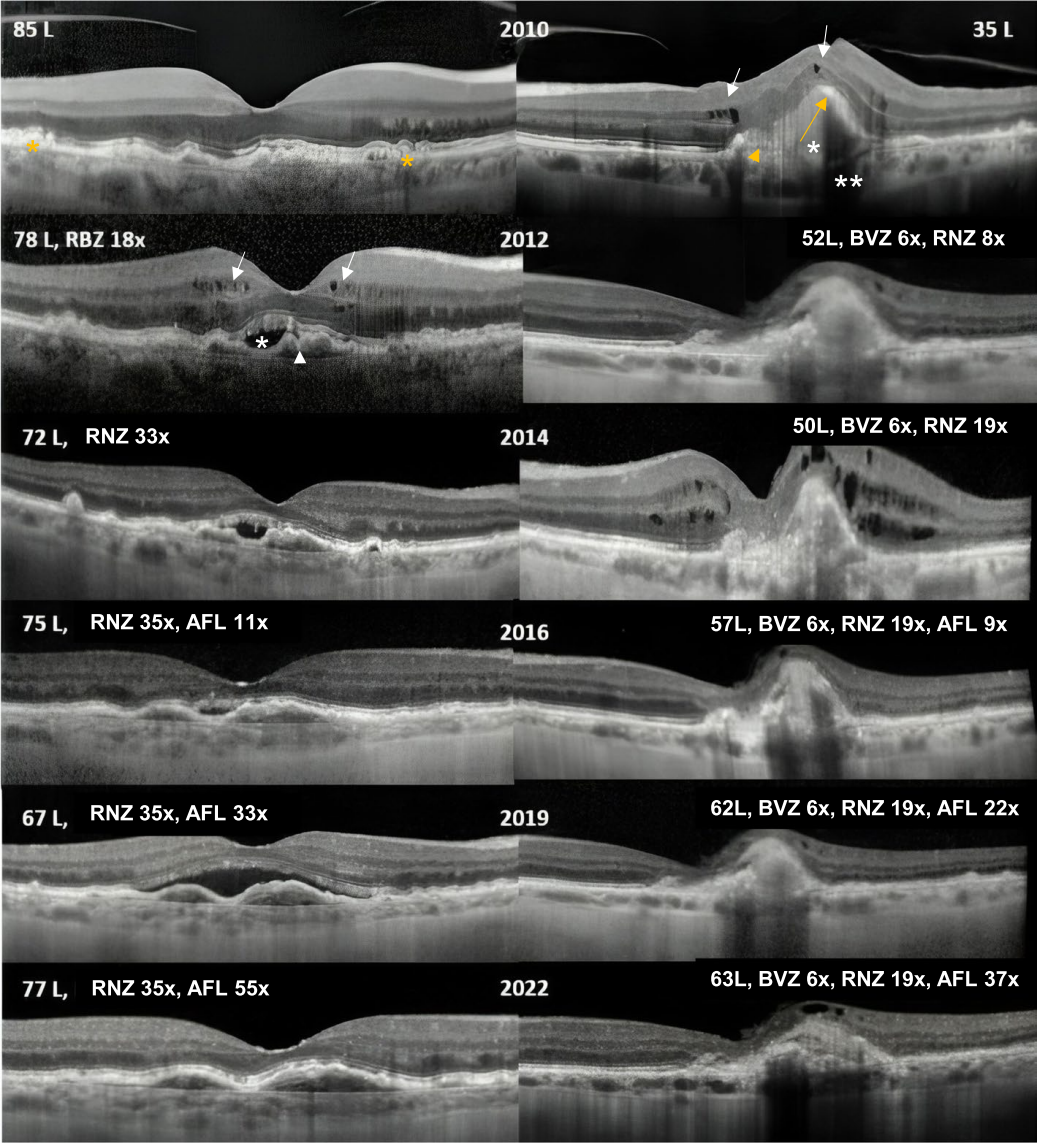
The OCT scan’s timeline for both eyes, right eye (RE) on the left side and left eye (LE) on the right side of the figure. The LE’s baseline OCT scan in 2010 suggests a PED (orange arrow) with loss of continuity of the RPE line (orange arrow head), a subretinal hyper-reflective area (asterisk) and intraretinal fluid (white arrows). There are transmission defects due to atrophy (double asterisk). The RE exhibits drusen (orange asterisk). However, within two months, the RE’s OCT showed intra (white arrows) and subretinal fluid (white asterisk) along with a flat PED (white arrowhead)

**Fig. 3:**
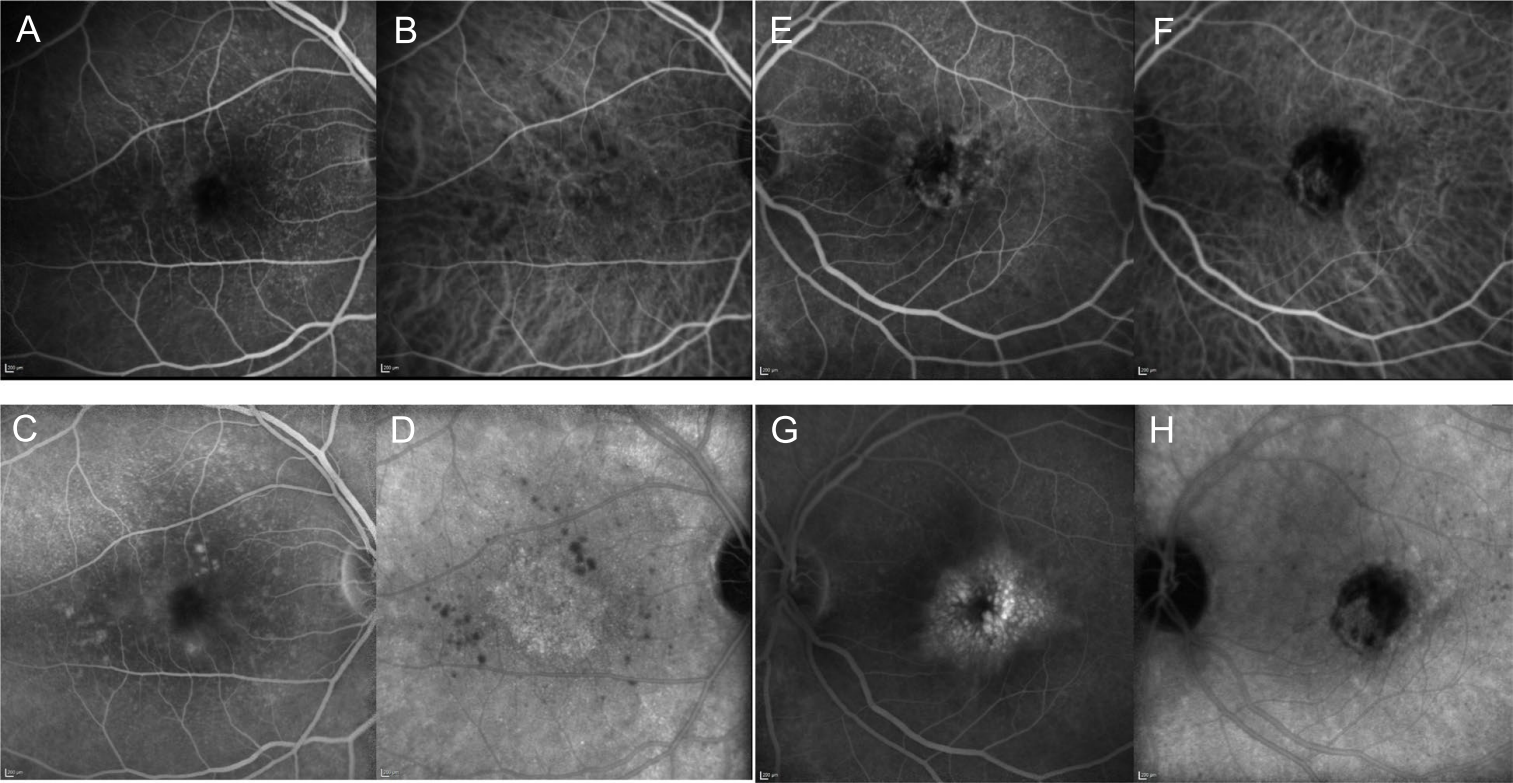
December 5, 2013’s FA and ICGA frames collected from the Spectralis device at 2 minutes (**A**, **B**, **E**, **F**) and at 20 minutes (**C**, **D**, **G**, **H**) of the RE (**A**, **B**, **C**, **D**) and the LE (**E**, **F**, **G**, **H**). Frames are paired with FA on the left side and ICGA on the right side. FA showed slight macular leakage at 2 minutes (**A**) increasing at 20 minutes in the RE (**C**), while ICGA revealed a clear, well delimited CNV in the late frame (**D**) whose boundaries were occult in its FA counterpart (**C**). In the LE there was intense and diffuse leakage at 2 minutes (**E**), increasing dramatically at 20 minutes assuming a cystoid apperance, together with mask and window defects (**G**). LE’s ICGA shows early central and peripheral ring-shaped hypercyanescence with a large mask effect (**F**) with late hypercyanescence pooling without matching FA hyperfluorescence (**H**), along with a mask effect that was considered to be related to a RPE tear

**Fig. 4:**
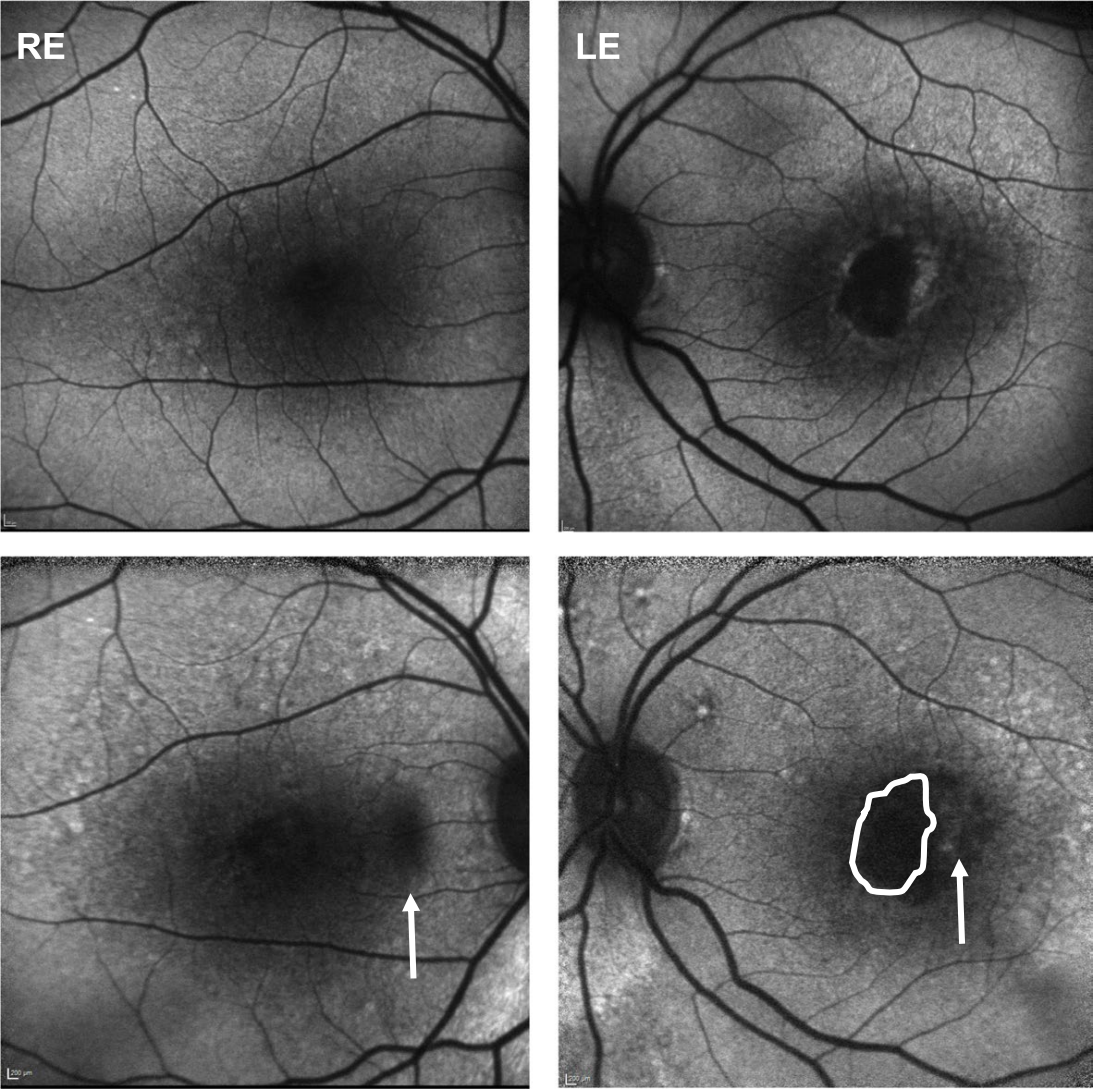
Fundus autofluorescence of the RE (left) and LE (right). Baseline frames (top) and 12-year’s frames (bottom). There is a slight progression of atrophy only in the RE after 12 years (hypoautofluorescent area, white arrow, left). Progression of atrophy is larger in the LE though (white arrow, right), where the encircled white area (bottom right) matches the drawn area of atrophy at baseline (top right)

At baseline, macular OCT of the LE showed intraretinal cysts, a RPE detachment and loss of continuity of the RPE line and a hyper-reflective subretinal lesion associated with fibrosis, loss of the ellipsoid zone and atrophy. RE’s OCT was unremarkable, apart from macular drusen, at first. However, intra-retinal cysts and subretinal fluid developed within two months (Fig. 2). Fluorescein angiography (FA) showed slight macular leakage at 2 min (') with increasing fluorescence at 20’ in the RE, and a more intense leakage at 2’ in the LE and intense fluorescence at 20’, together with mask and window defects. Indocyanin green angiography (ICGA) revealed at 20’ a clear, well delimited CNV in the RE unseen in FA, and late hypercyanescence of the LE that imperfectly matched FA hyperfluorescence along with a mask effect that was considered to be related to a RPE tear. A diagnosis of an occult lesion or Type 1 CNV in the RE and a minimally classic/classic lesion or Type 2 CNV in the LE was established (Fig. 3). Fundus autofluorescence (FAF) revealed a central area of hypoautofluorescence in the RE that was attributed to the presence of lutein with focal areas of hypo/hyperautofluorescence releated to drusen and RPE mottling. LE’s FAF revealed a central area of hypoautofluorescence surrounded by a ring of hyperautofluorescence that was related to the RPE tear. FAF at the end of the study showed a slight progression of atrophy, in the LE mainly (Fig. 4).

## Results

Therapy began with nine injections of bevacizumab (*n* = 6) and ranibizumab (*n* = 3) in the LE within the first year of treatment. Treatment of the RE followed, with a total of seven injections of ranibizumab within the first year. The RE received a mean of 7.5 injections per year, with a total of 90 injections, 35 of ranibizumab and 55 of aflibercept. The LE had a mean of 5.2 injections/year with a total of 62 injections in 12 years, 6 of bevacizumab, 19 of ranibizumab and 37 of aflibercept (Table 1). Meanwhile, she underwent cataract surgery on 2021.

**Table 1:** Number of injections/year in the follow-up time

T-Year	1^st^	2^nd^	3^rd^	4^th^	5^th^	6^th^	7^th^	8^th^	9^th^	10^th^	11^th^	12^th^	Total
RE	7	8	7	7	7	6	8	8	8	9	9	6	90
LE	9	5	5	5	5	5	5	5	5	5	4	4	62
Year	2010–2011					2015–2016						2021–2022	

*T*-Year, year of treatment, *RE*, right eye, *LE*, left eye. Baseline for the *RE* was August 25, 2010 and *LE’s* was June 2, 2010. Each and every year of treatment was counted back from those dates

**Fig. 5:**
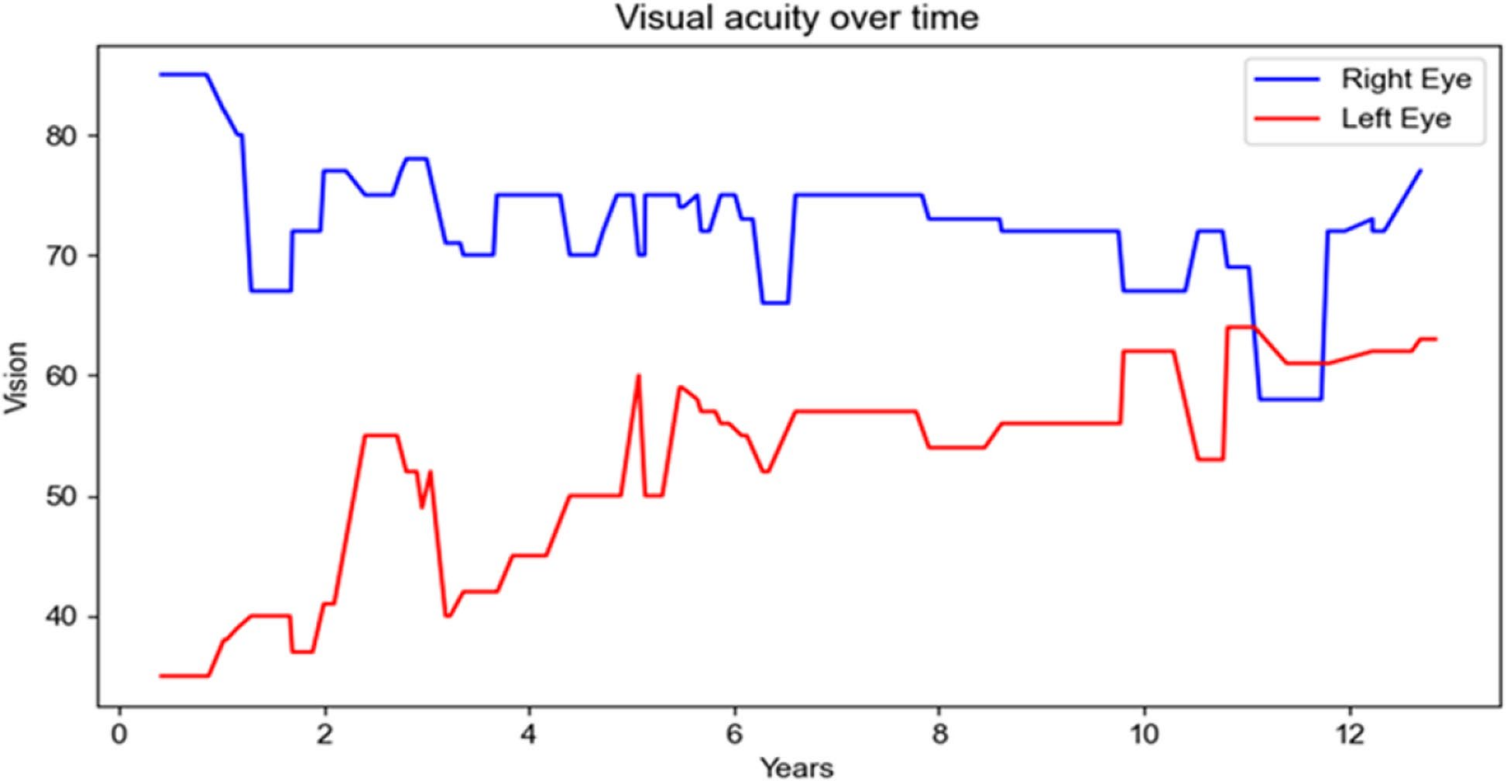
Timeline of BCVA. Blue line for the right eye and red line for the left eye. From baseline in 2010, each subsequent year corresponds to the subsequent numbering. In 2010, the LE had 35L and the RE had 85L. They ended up in 2022 with 63L and 77L, respectively

**Fig. 6:**
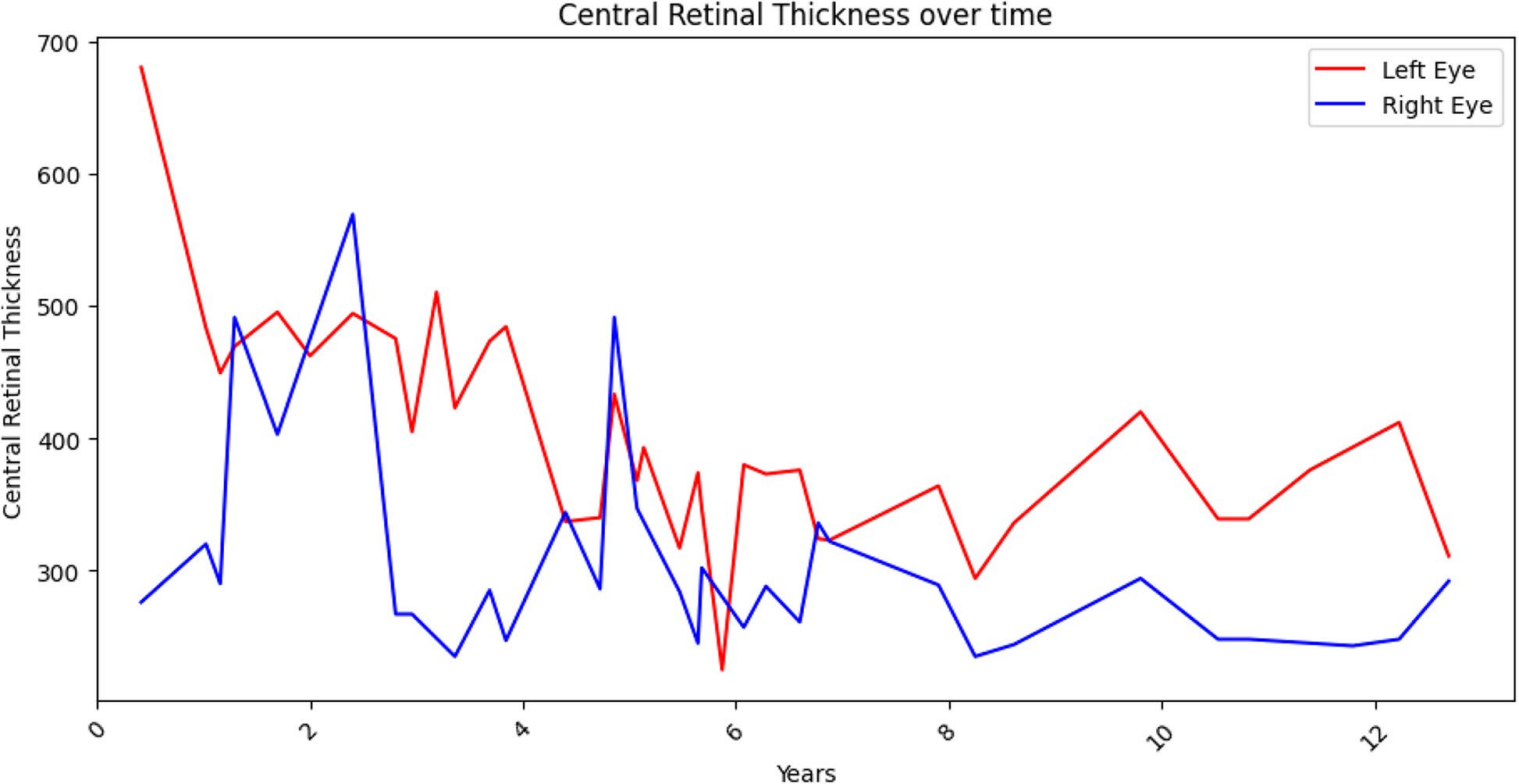
Timeline of the CRT of either eye. Blue line for the right eye and red line for the left eye. At baseline, the LE had a CRT of 680 μm, while the RE had 276 μm. There was some fluctuation along the follow-up time despite treatment

LE’s BCVA improved up to 38L (+ 3L) in six months (Fig. 5) and reached a plateau after the first five years of treatment, up to 58L (+ 23L from baseline). Nevertheless, there has been a significant decrease in the BCVA related to cataract in year eleven. After cataract removal LE’s final BCVA was 63L (+ 28L from baseline). LE’s mean central retinal thickness (CRT) decreased from 680 μm to 483 μm during the first year of treatment and continued to show a significant decrease, followed by a continued reduction until year six, to its minimum of 311 μm (Fig. 6). Overall, LE’s BCVA increased 28L (from baseline 35L to 63L in year 12) and CRT decreased 369 μm (from 680 μm at presentation to 311 μm in year twelfth).

Conversely, the BCVA of the RE initially declined (during the first two years), from baseline 85L to 67L (Fig. 5). Thereafter, it reached a plateau, between 70 and 75L. There was a sudden drop to 58L during early 2021, followed by a quick recovery, related to cataract and cataract surgery. The CRT was not steady during the first two years of treatment and showed a late increase at year five. However, it remained stable afterwards (Fig. 6). RE’s BCVA dropped 8L along the 12-year timeline (85L to 77L, Fig. 5). The difference in CRT along the 12-year timeline was minimal (from baseline 276 μm to 292 μm in the twelfth year), Fig. 6 and Supplementary file.

## Discussion/conclusion

This case report presents a 12-year follow-up of a naïve patient with nAMD who recovered BCVA from the worst eye (+ 28L, LE 63L, ≈20/50 or reading vision) and kept driving vision in the best eye (-8L, RE 77L, ≈ 20/25), with a mean of 7.5 injections per year in the RE and 5.2 injections in the LE. Data from this study are in line with the findings that baseline BCVA is the paramount prognostic factor for the final BCVA in nAMD. The compliance of the patient was excellent, our department has a system of calling back missing patients and our team did not stop the intravitreal treatments during the COVID pandemic at all [9]. Along the timeline we started with a *pro re nata* (PRN) protocol, changed for treat and extend (T&E) in 2016 and adopted a fixed protocol in 2020 with more injections in the first semester of the first year than in the second [9]. Since the adjustments of the T&E regimen are based in the presence or absence of relapses, it is not surprising that intra-retinal or subretinal fluid is present in OCT scans until 2019 (Fig. 2). Noteworthy, injections were more frequent in the first year, since the LE had 7 injections in the first year of treatment and the RE had 9 injections (Table 1). It has been reported that the outcomes are primarily related to the number of injections and these numbers are not far from the “magic” number of 8 injections in the first year [3–5, 9]. Moreover, the fixed regimen decreased the number of required visits from 8 to 2 visits/year, since the OCT scan was always made in the same day of the injection [6, 9].

It is known that eyes with lower baseline BCVA do not reach a final BCVA as high as eyes with higher baseline BCVA. Nevertheless, they may recover more letters, since they do not suffer from ceiling effects [10]. CNV subtype 1 is related to better prognosis, however some authors relate lesion size (and time to starting treatment) and not subtype to prognosis [11]. Time to treatment or delay has been related to worse final outcome with the development of atrophy, development of more complex lesions and loss of the ellipsoid zone, as happened in our patient’s LE [12]. Nevertheless, in this case report everything fitted with these data: the eye with the worst visual acuity at onset recovered more letters but did not get a final visual acuity as its fellow eye. Moreover, it had a CNV subtype associated with a worse prognosis and took longer to be treated [11]. At the end of the day, this case report does not focus on the different outcomes of either eye, but on the fact that both kept a good visual acuity after twelve years, of driving or reading, respectively, seldom seen in nAMD, where real-world data indicate a progressive loss of visual acuity [4, 13]. We think this was associated with a more proactive treatment approach. It is known that nAMD treatment results fall short from the desired, either in reported series and Medicare claims data [5]. The need for more proactive approaches and the value of a fixed regimen were recently reported [5, 8]. We think that the fixed approach based on the number of injections, OCT on the day of injection and a protocol of calling back missing patients may help to improve the final results in a considerable number of patients. The fixed regimen has a minimum number of injections per year and allows, if necessary, an extra injection at any time. It is not reactive, since it is not based on relapses and it consistently stands on the the number of injections given, the best evidence for treatment outcomes in nAMD [3, 4]. Additionally, it spares a lot of visits (from 15 to 4 within a 2-year period) if the OCT scan is made on the day of injection [6, 9].

Our study has limitations. Most prominently, it is a case report and the results cannot be extrapolated. The LE was treated with bevacizumab, ranibizumab and aflibercept, which are different anti-VEGF agents. However, the aim of this study was not to compare the outcomes between eyes, but to report that BCVA may be preserved in either eye in the long run if we do have a proactive approach. Additionally, data from the CATT study showed that there are no meaningful differences between the two drugs at the end of the first year in AMD [14]. As a strength, this work may have an exploratory value. The fixed protocols may have similar or better results than the PRN or T&E approaches, with a slight trend to overtreatment, albeit with fewer visits. The approaches of the newer drugs seem to encourage the fixed approach [15–17].

In conclusion, though the final visual outcome depends on baseline BCVA and lesion type or size, the number of injections is paramount in preserving BCVA and achieving favorable functional outcomes in nAMD, even after 12 years of treatment.

### Supplementary Information

**Supplementary Material 1. **

## Data Availability

The data are accessible through Retina.PT, a platform hosted on the website https://www.ger-portugal.com/. However, since accessing the data requires an account login, please let us know how we can assist you further with any specific questions or tasks related to data analyses.
