# Supplementary material for: Preserving visual acuity: a compelling 12-year case study of controlling neovascular age-related macular degeneration
Source: BMC Ophthalmol. 2024 Mar 18;24:123. doi: 10.1186/s12886-024-03387-9 (PMC10946101; doi:10.1186/s12886-024-03387-9)
Supplement: Supplementary file 1 — Supplementary Material 1. [file 12886_2024_3387_MOESM1_ESM.pdf]

OCT Thickness Map Changes

January 2024

1 Right eye

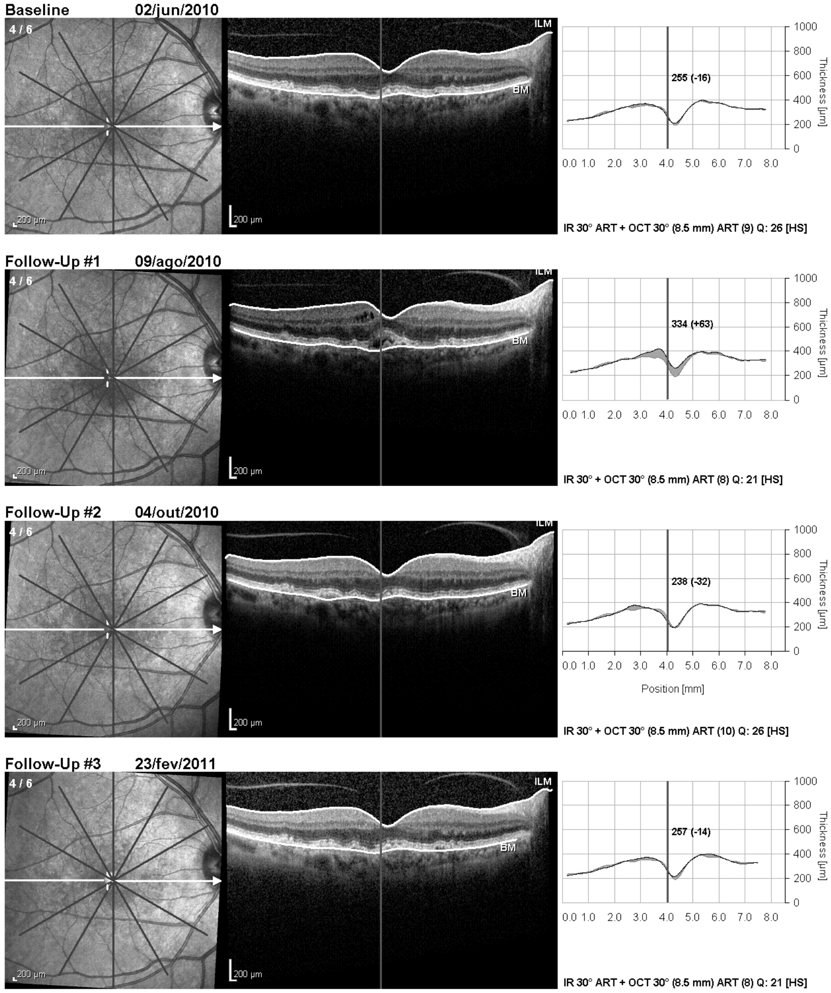
Baseline 02/jun/2010

255 (-16)

IR 30° ART + OCT 30° (8.5 mm) ART (9) Q: 26 [HS]

Follow-Up #1 09/ago/2010

334 (+63)

IR 30° + OCT 30° (8.5 mm) ART (8) Q: 21 [HS]

Follow-Up #2 04/out/2010

238 (-32)

Position [mm]

IR 30° + OCT 30° (8.5 mm) ART (10) Q: 26 [HS]

Follow-Up #3 23/fev/2011

257 (-14)

IR 30° ART + OCT 30° (8.5 mm) ART (8) Q: 21 [HS]

Notes:

Date: 03/01/2024 Signature:

Software Version: 6.12.4 www.HeidelbergEngineering.com Retina Change Report, All Follow-Ups, Page 1/7

Follow-Up #4 **27/set/2011**

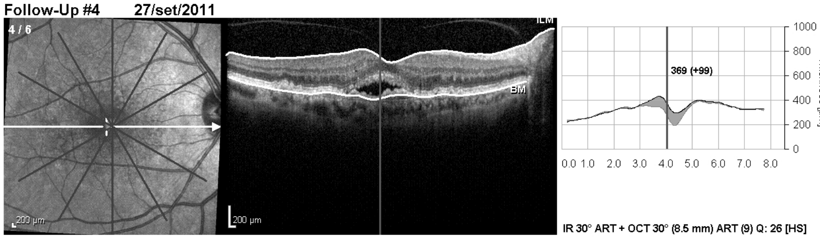

IR 30° ART + OCT 30° (8.5 mm) ART (9) Q: 26 [HS]

Follow-Up #5 **20/out/2011**

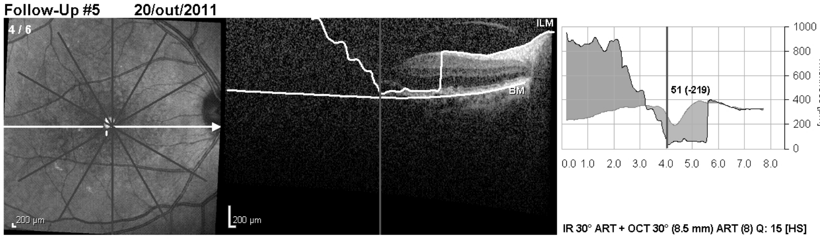

IR 30° ART + OCT 30° (8.5 mm) ART (8) Q: 15 [HS]

Follow-Up #6 **22/nov/2011**

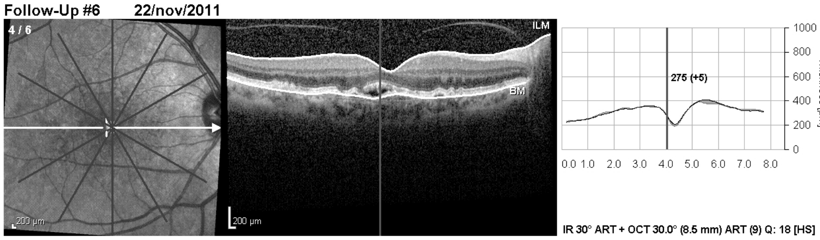

IR 30° ART + OCT 30.0° (8.5 mm) ART (9) Q: 18 [HS]

Follow-Up #7 **20/dez/2011**

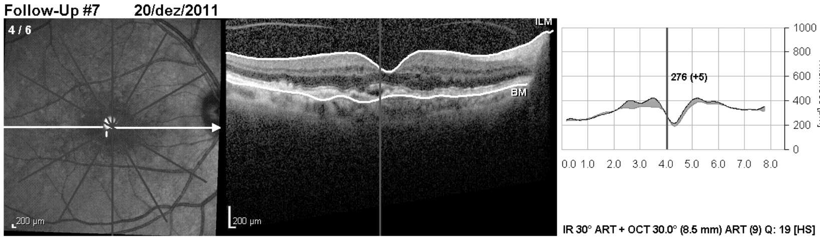

IR 30° ART + OCT 30.0° (8.5 mm) ART (9) Q: 19 [HS]

Notes:

Date: 03/01/2024 Signature:

[illegible]

Follow-Up #8 17/abr/2012

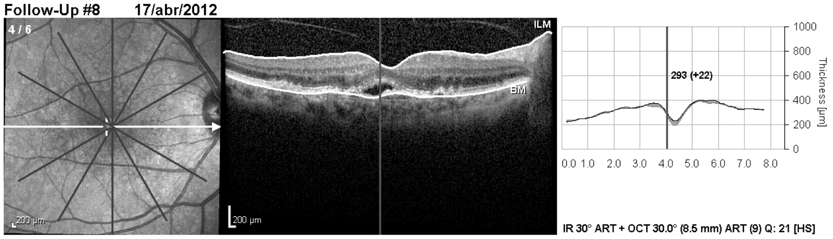

IR 30° ART + OCT 30.0° (8.5 mm) ART (9) Q: 21 [HS]

Follow-Up #9 25/mai/2012

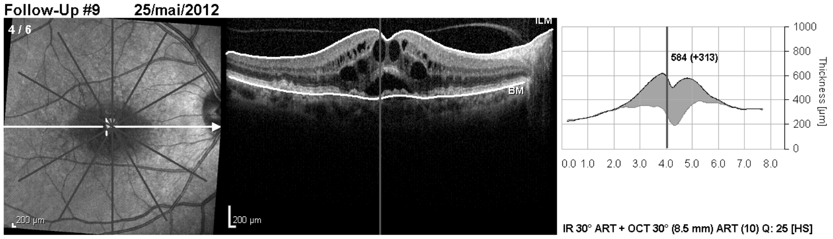

IR 30° ART + OCT 30° (8.5 mm) ART (10) Q: 25 [HS]

Follow-Up #10 12/jun/2012

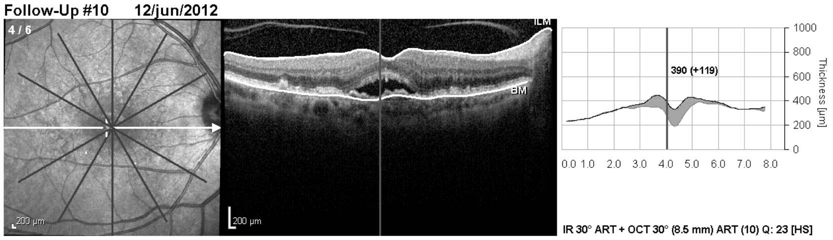

IR 30° ART + OCT 30° (8.5 mm) ART (10) Q: 23 [HS]

Follow-Up #11 13/jul/2012

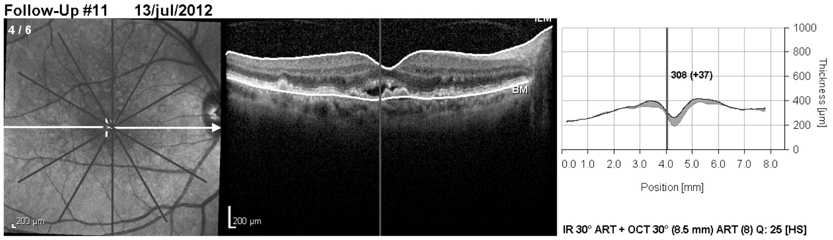

IR 30° ART + OCT 30° (8.5 mm) ART (8) Q: 25 [HS]

Notes:

Date: 03/01/2024 Signature:

Follow-Up #12 31/ago/2012

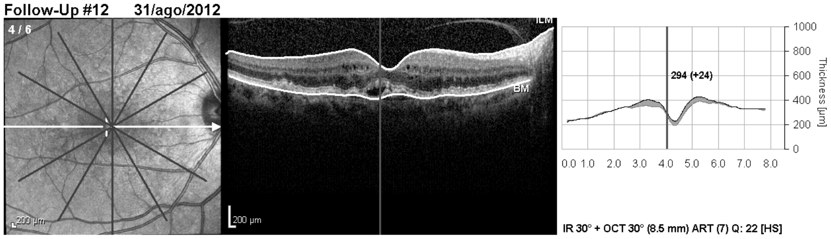

IR 30° + OCT 30° (8.5 mm) ART (7) Q: 22 [HS]

Follow-Up #13 24/set/2012

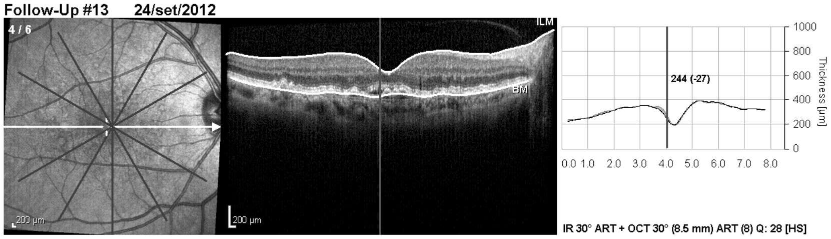

IR 30° ART + OCT 30° (8.5 mm) ART (8) Q: 28 [HS]

Follow-Up #14 19/out/2012

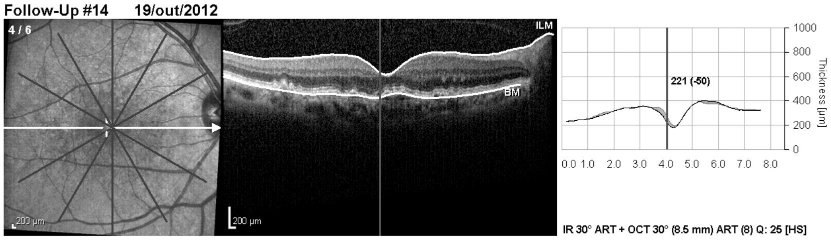

IR 30° ART + OCT 30° (8.5 mm) ART (8) Q: 25 [HS]

Follow-Up #15 02/nov/2012

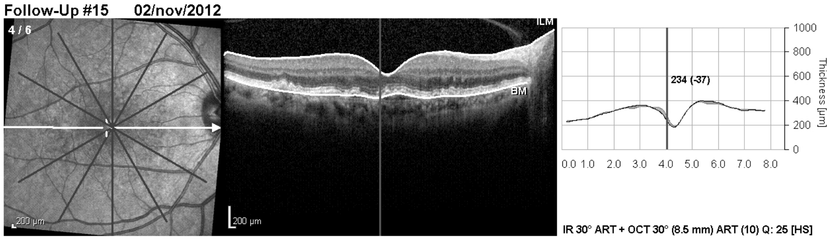

IR 30° ART + OCT 30° (8.5 mm) ART (10) Q: 25 [HS]

Notes:	
Date: 03/01/2024	Signature:

[illegible]

Follow-Up #16 **11/dez/2012**

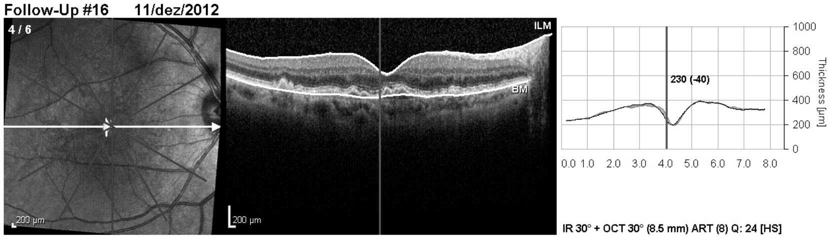

IR 30° + OCT 30° (8.5 mm) ART (8) Q: 24 [HS]

Follow-Up #17 **14/jan/2013**

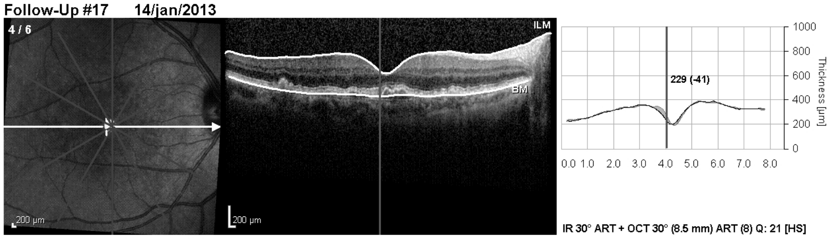

IR 30° ART + OCT 30° (8.5 mm) ART (8) Q: 21 [HS]

Follow-Up #18 **11/mar/2013**

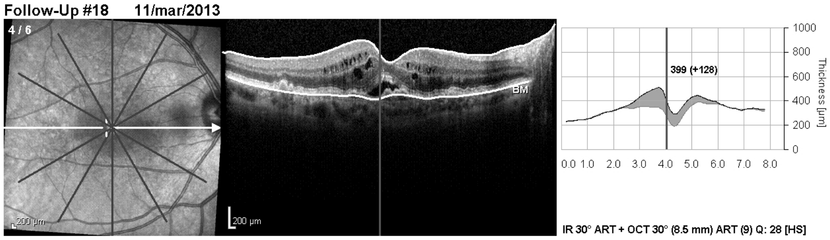

IR 30° ART + OCT 30° (8.5 mm) ART (9) Q: 28 [HS]

Follow-Up #19 **12/abr/2013**

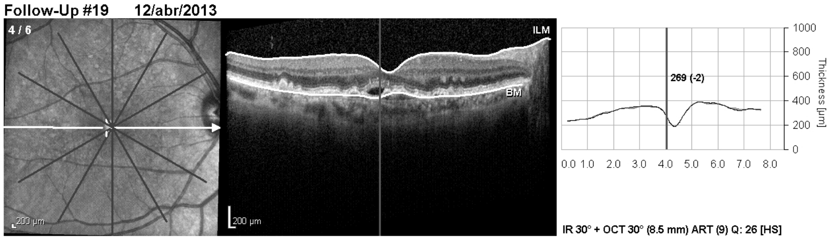

IR 30° + OCT 30° (8.5 mm) ART (9) Q: 26 [HS]

Notes:

Date: 03/01/2024 Signature:

Follow-Up #20 13/mai/2013

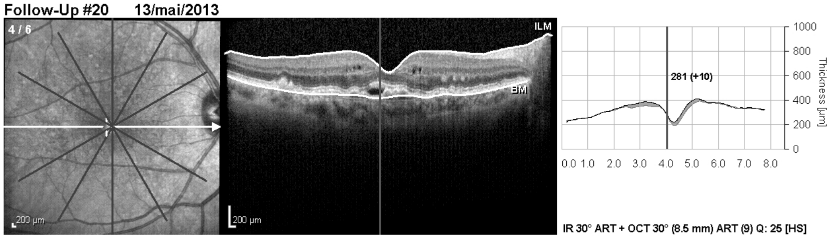

IR 30° ART + OCT 30° (8.5 mm) ART (9) Q: 25 [HS]

Follow-Up #21 25/jun/2013

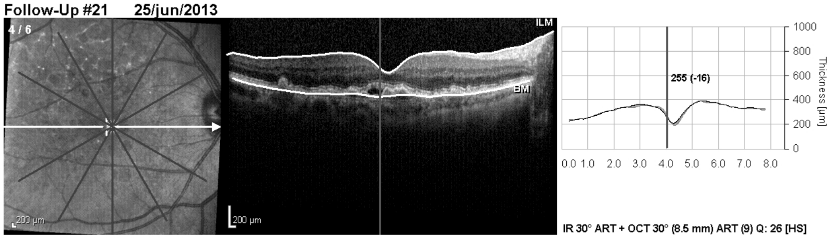

IR 30° ART + OCT 30° (8.5 mm) ART (9) Q: 26 [HS]

Follow-Up #22 26/ago/2013

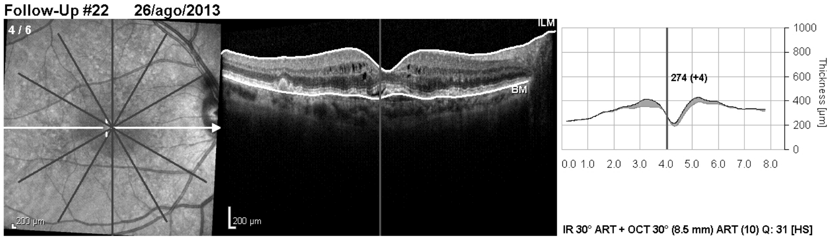

IR 30° ART + OCT 30° (8.5 mm) ART (10) Q: 31 [HS]

Follow-Up #23 02/set/2013

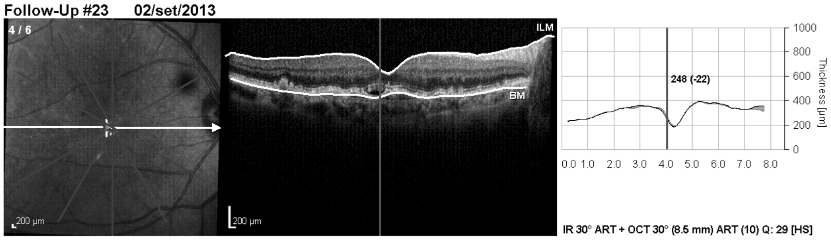

IR 30° ART + OCT 30° (8.5 mm) ART (10) Q: 29 [HS]

Notes:	
Date: 03/01/2024	Signature:

[illegible]

Follow-Up #24 **01/out/2013**

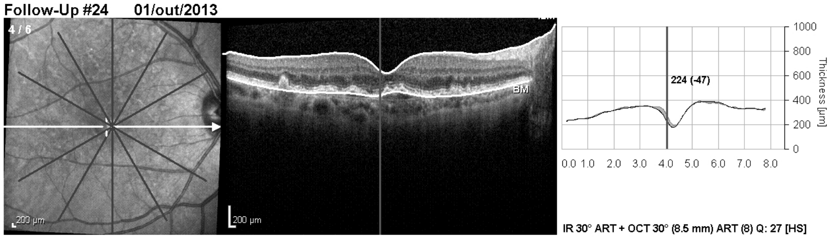

IR 30° ART + OCT 30° (8.5 mm) ART (8) Q: 27 [HS]

Reference **04/nov/2013**

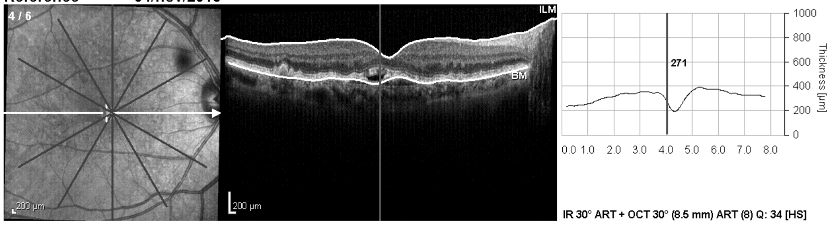

IR 30° ART + OCT 30° (8.5 mm) ART (8) Q: 34 [HS]

Follow-Up #26 **10/jan/2014**

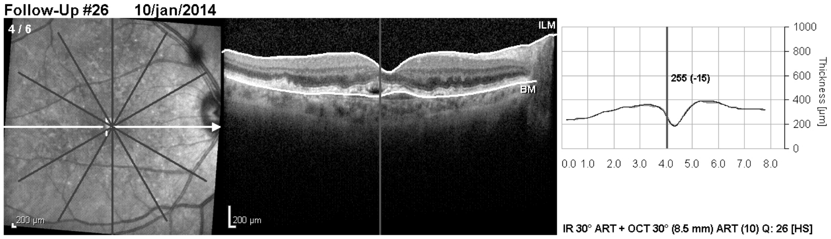

IR 30° ART + OCT 30° (8.5 mm) ART (10) Q: 26 [HS]

Notes:	
Date: 03/01/2024	Signature:

Baseline **10/jan/2014**

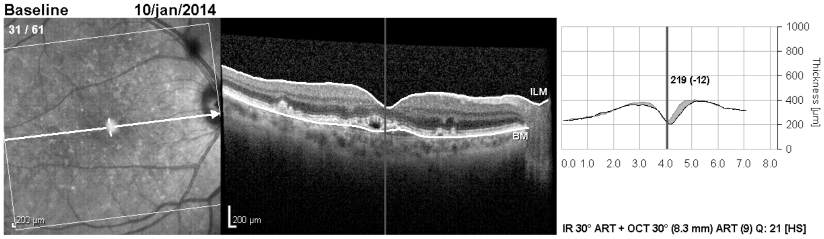

IR 30° ART + OCT 30° (8.3 mm) ART (9) Q: 21 [HS]

Follow-Up #1 **21/fev/2014**

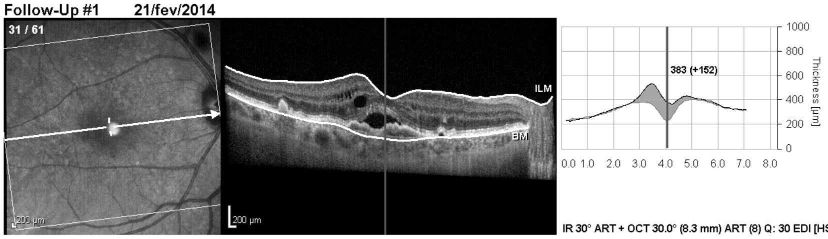

IR 30° ART + OCT 30.0° (8.3 mm) ART (8) Q: 30 EDI [HS]

Follow-Up #2 **19/mar/2014**

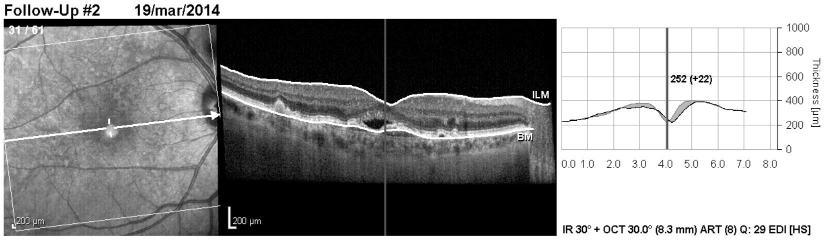

IR 30° + OCT 30.0° (8.3 mm) ART (8) Q: 29 EDI [HS]

Follow-Up #3 **22/abr/2014**

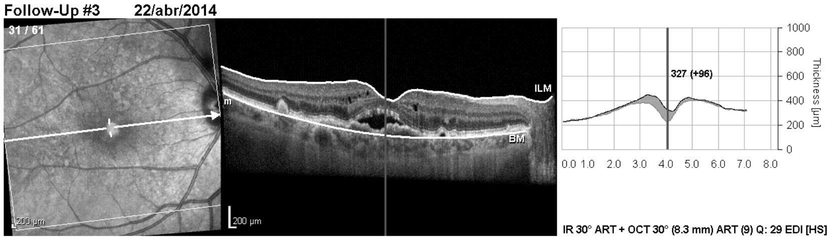

IR 30° ART + OCT 30° (8.3 mm) ART (9) Q: 29 EDI [HS]

Notes:

Date: 03/01/2024 Signature:

Software Version: 6.12.4 *www.HeidelbergEngineering.com* *Retina Change Report, All Follow-Ups, Page 1/9*

Follow-Up #4 **17/jun/2014**

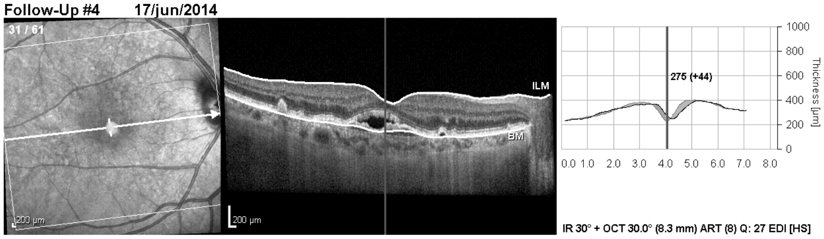

IR 30° + OCT 30.0° (8.3 mm) ART (8) Q: 27 EDI [HS]

Follow-Up #5 **02/jul/2014**

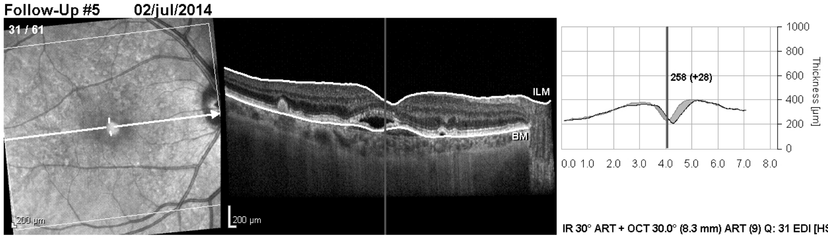

IR 30° ART + OCT 30.0° (8.3 mm) ART (9) Q: 31 EDI [HS]

Follow-Up #6 **23/set/2014**

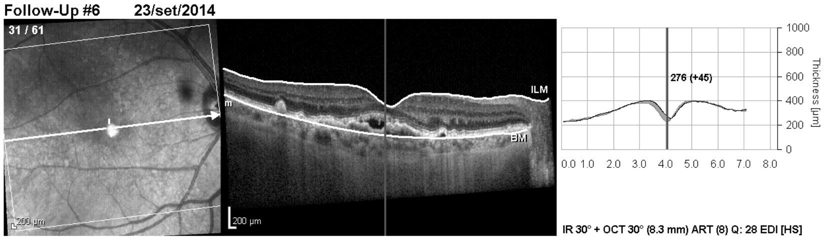

IR 30° + OCT 30° (8.3 mm) ART (8) Q: 28 EDI [HS]

Follow-Up #7 **11/nov/2014**

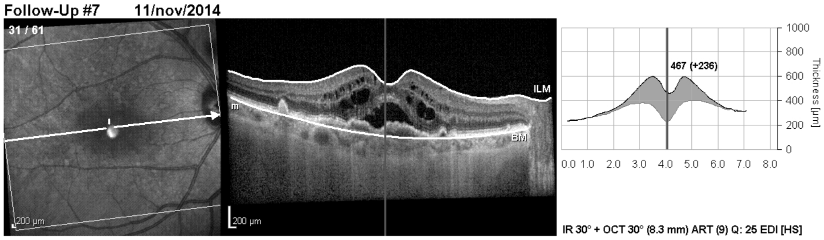

IR 30° + OCT 30° (8.3 mm) ART (9) Q: 25 EDI [HS]

Notes:	
Date: 03/01/2024	Signature:

[illegible]

Follow-Up #8 **15/dez/2014**

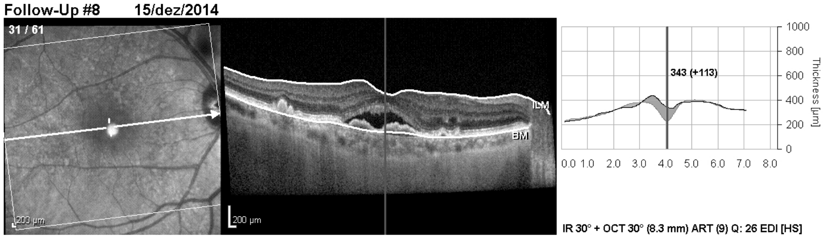

IR 30° + OCT 30° (8.3 mm) ART (9) Q: 26 EDI [HS]

Follow-Up #9 **26/jan/2015**

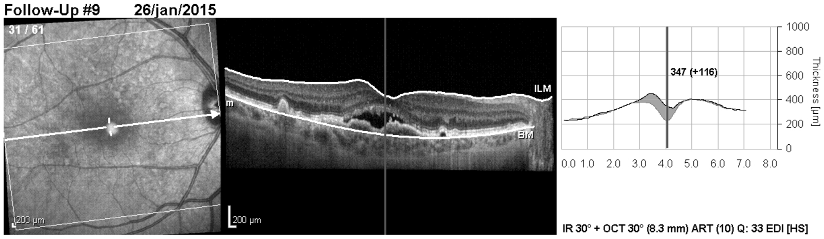

IR 30° + OCT 30° (8.3 mm) ART (10) Q: 33 EDI [HS]

Follow-Up #10 **20/fev/2015**

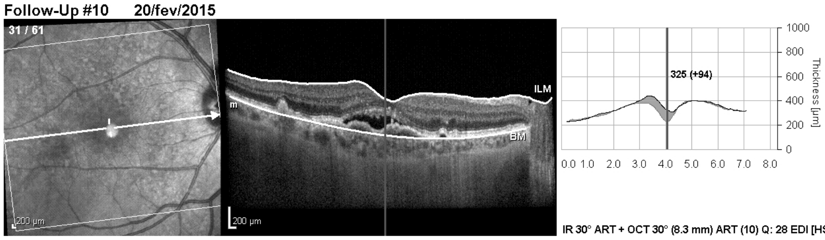

IR 30° ART + OCT 30° (8.3 mm) ART (10) Q: 28 EDI [HS]

Follow-Up #11 **24/mar/2015**

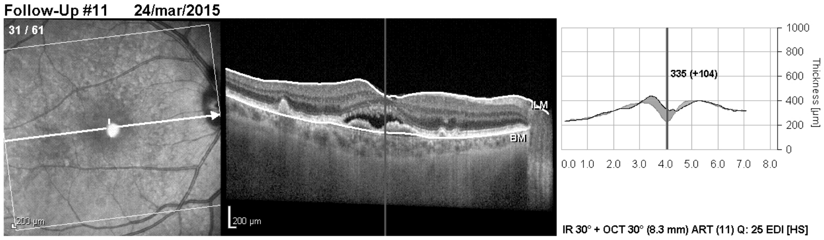

IR 30° + OCT 30° (8.3 mm) ART (11) Q: 25 EDI [HS]

Notes:

Date: 03/01/2024 Signature:

Follow-Up #12 11/mai/2015

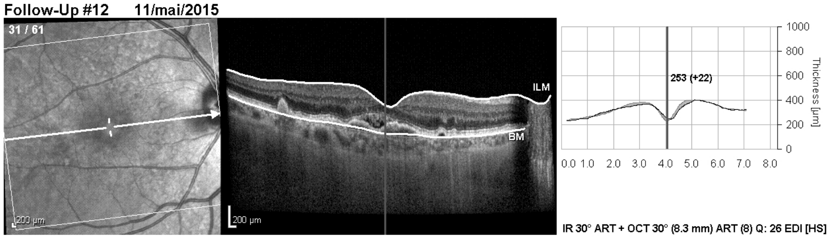

IR 30° ART + OCT 30° (8.3 mm) ART (8) Q: 26 EDI [HS]

Follow-Up #13 03/jun/2015

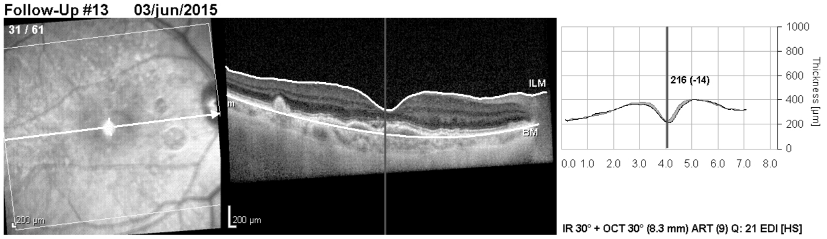

IR 30° + OCT 30° (8.3 mm) ART (9) Q: 21 EDI [HS]

Follow-Up #14 01/jul/2015

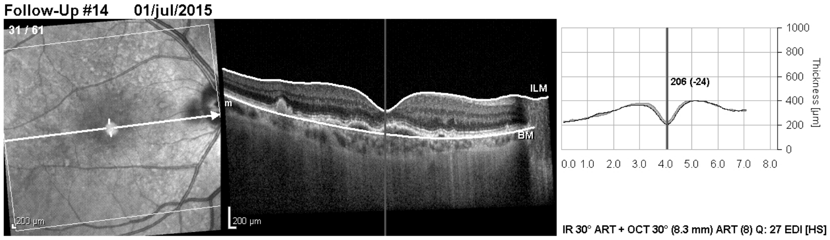

IR 30° ART + OCT 30° (8.3 mm) ART (8) Q: 27 EDI [HS]

Follow-Up #15 05/ago/2015

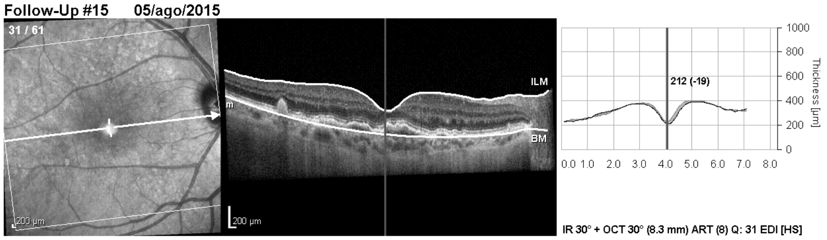

IR 30° + OCT 30° (8.3 mm) ART (8) Q: 31 EDI [HS]

Notes:

Date: 03/01/2024   Signature:

[illegible]

Follow-Up #16 29/set/2015

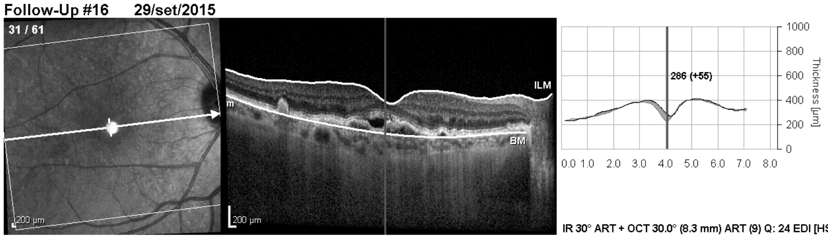

IR 30° ART + OCT 30.0° (8.3 mm) ART (9) Q: 24 EDI [HS]

Follow-Up #17 09/dez/2015

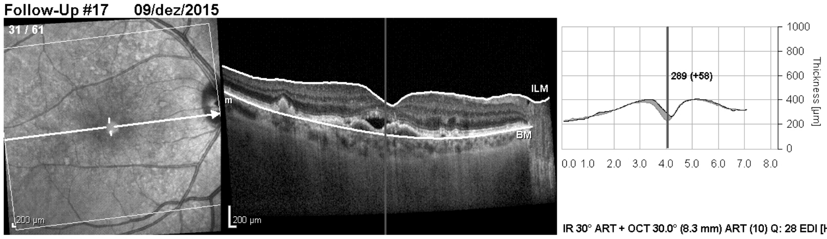

IR 30° ART + OCT 30.0° (8.3 mm) ART (10) Q: 28 EDI [HS]

Follow-Up #18 14/jan/2016

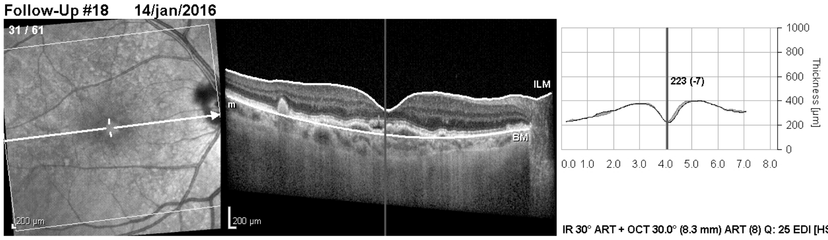

IR 30° ART + OCT 30.0° (8.3 mm) ART (8) Q: 25 EDI [HS]

Follow-Up #19 09/mar/2016

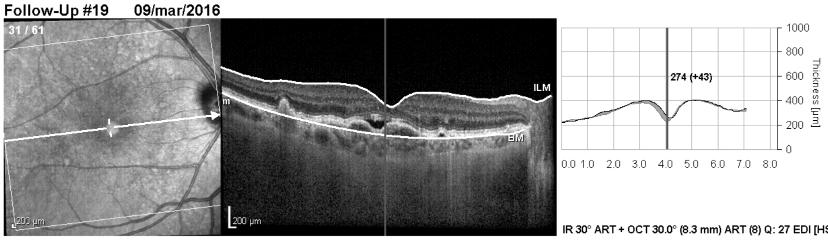

IR 30° ART + OCT 30.0° (8.3 mm) ART (8) Q: 27 EDI [HS]

Notes:

Date: 03/01/2024 Signature:

[illegible]

Follow-Up #20 **04/mai/2016**

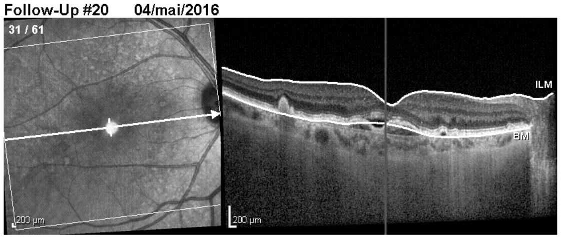

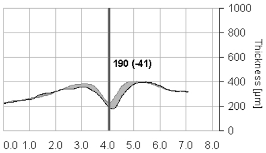

IR 30° ART + OCT 30.0° (8.3 mm) ART (10) Q: 24 EDI [HS]

Follow-Up #21 **05/ago/2016**

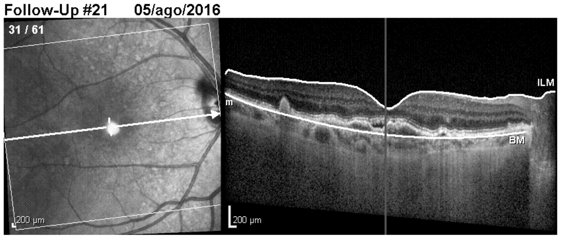

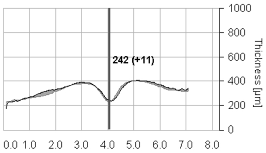

IR 30° ART + OCT 30° (8.3 mm) ART (9) Q: 24 EDI [HS]

Follow-Up #22 **17/out/2016**

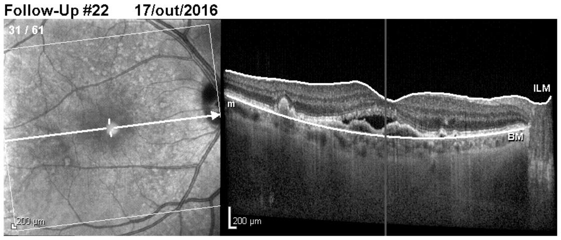

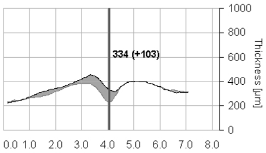

IR 30° ART + OCT 30° (8.3 mm) ART (10) Q: 26 EDI [HS]

Follow-Up #23 **28/nov/2016**

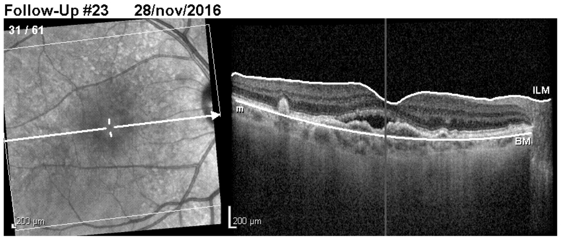

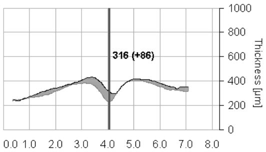

IR 30° ART + OCT 30° (8.3 mm) ART (8) Q: 30 EDI [HS]

Notes:
Date: 03/01/2024 Signature:

Follow-Up #4 28/nov/2016

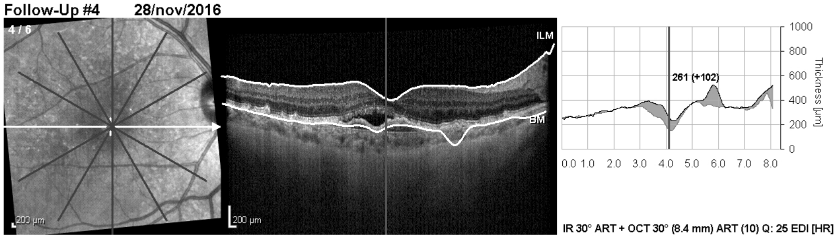

IR 30° ART + OCT 30° (8.4 mm) ART (10) Q: 25 EDI [HR]

Follow-Up #5 27/fev/2017

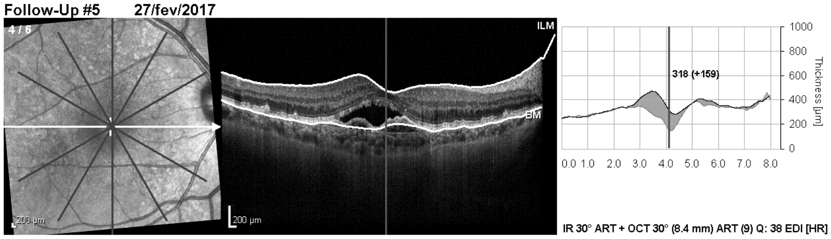

IR 30° ART + OCT 30° (8.4 mm) ART (9) Q: 38 EDI [HR]

Follow-Up #6 05/mai/2017

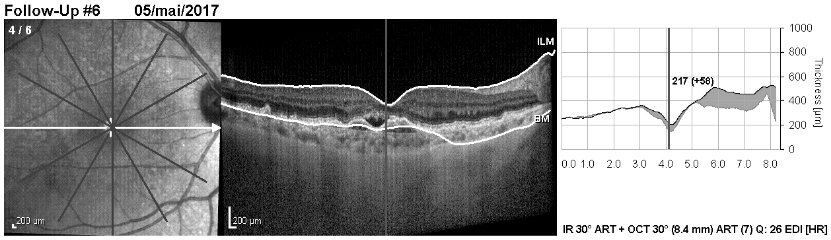

IR 30° ART + OCT 30° (8.4 mm) ART (7) Q: 26 EDI [HR]

Follow-Up #7 14/jul/2017

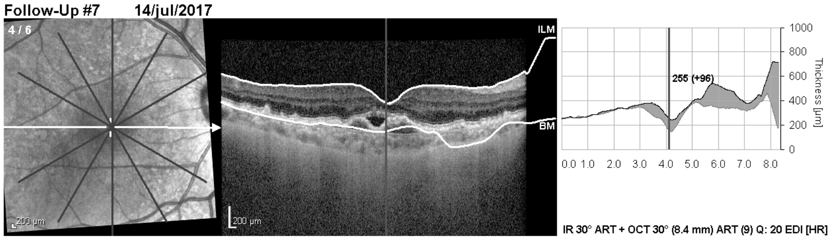

IR 30° ART + OCT 30° (8.4 mm) ART (9) Q: 20 EDI [HR]

Notes:

Date: 03/01/2024 Signature:

Diagnosis: Comment:

Follow-Up #8 05/set/2017

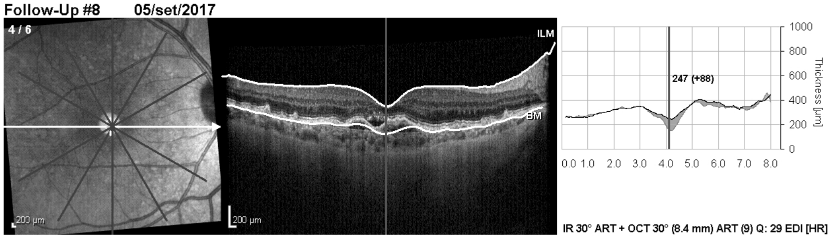

IR 30° ART + OCT 30° (8.4 mm) ART (9) Q: 29 EDI [HR]

Follow-Up #9 27/nov/2017

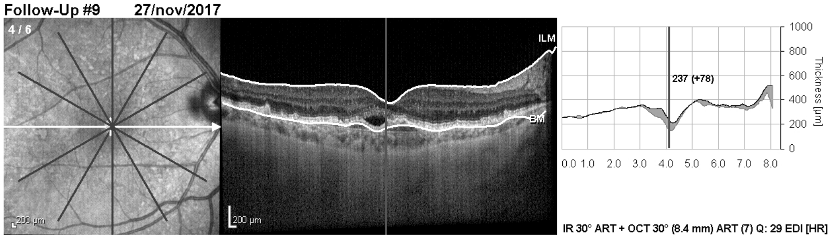

IR 30° ART + OCT 30° (8.4 mm) ART (7) Q: 29 EDI [HR]

Follow-Up #10 16/jan/2018

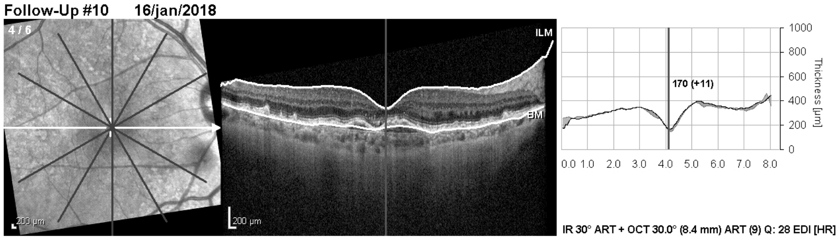

IR 30° ART + OCT 30.0° (8.4 mm) ART (9) Q: 28 EDI [HR]

Follow-Up #11 08/mar/2018

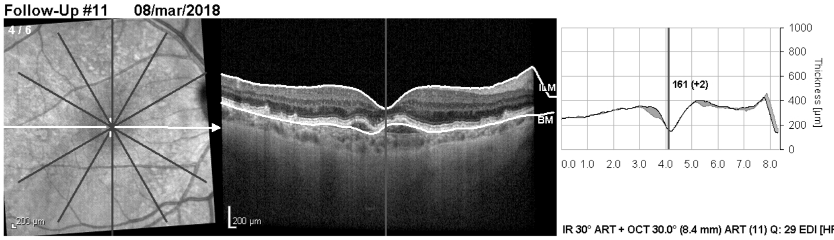

IR 30° ART + OCT 30.0° (8.4 mm) ART (11) Q: 29 EDI [HR]

Notes:

Date: 03/01/2024 Signature:

Follow-Up #12 **02/abr/2018**

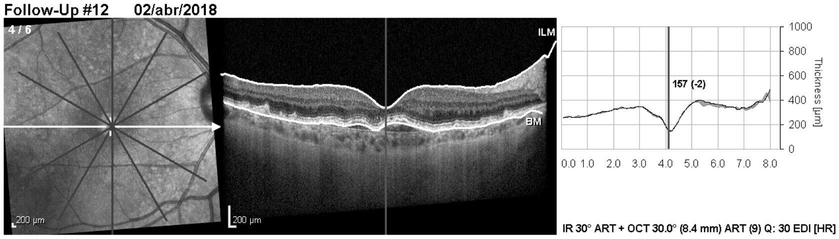

IR 30° ART + OCT 30.0° (8.4 mm) ART (9) Q: 30 EDI [HR]

Follow-Up #13 **05/jun/2018**

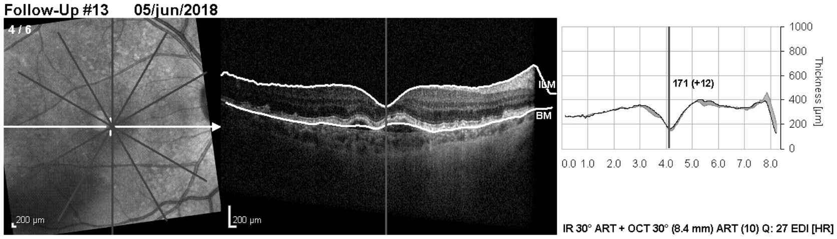

IR 30° ART + OCT 30° (8.4 mm) ART (10) Q: 27 EDI [HR]

Follow-Up #14 **13/ago/2018**

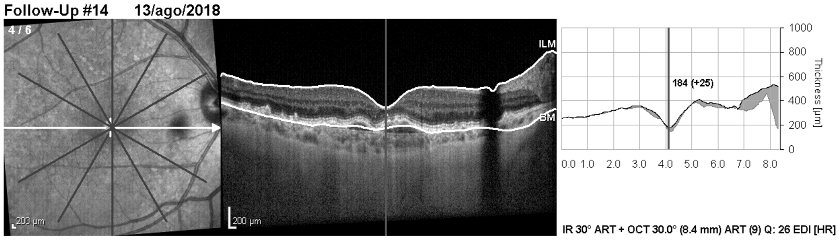

IR 30° ART + OCT 30.0° (8.4 mm) ART (9) Q: 26 EDI [HR]

Follow-Up #15 **20/nov/2018**

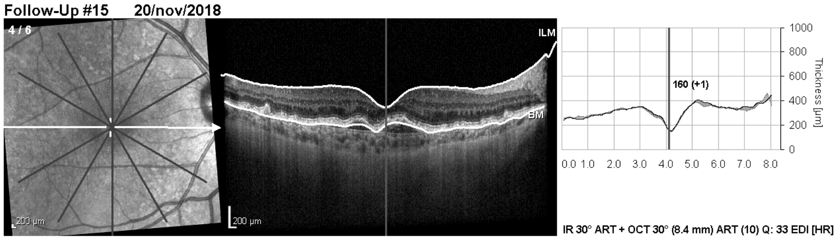

IR 30° ART + OCT 30° (8.4 mm) ART (10) Q: 33 EDI [HR]

Notes:

Date: 03/01/2024 Signature:

Follow-Up #16 **04/fev/2019**

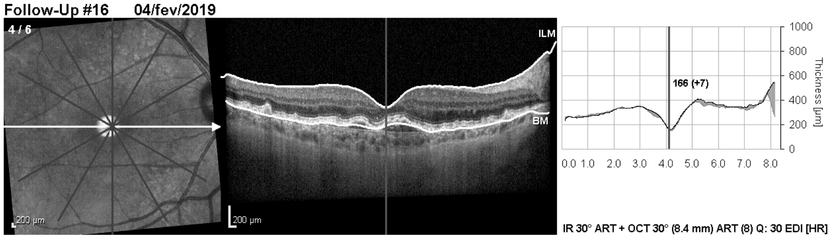

IR 30° ART + OCT 30° (8.4 mm) ART (8) Q: 30 EDI [HR]

Follow-Up #17 **15/abr/2019**

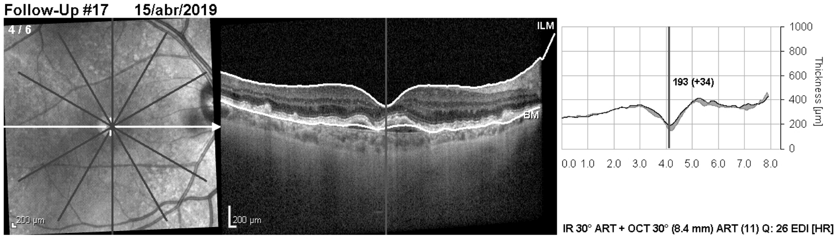

IR 30° ART + OCT 30° (8.4 mm) ART (11) Q: 26 EDI [HR]

Follow-Up #18 **15/mai/2019**

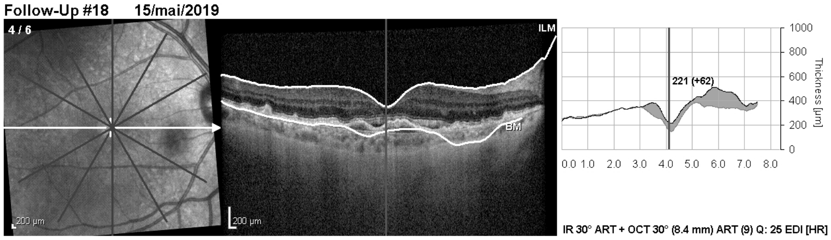

IR 30° ART + OCT 30° (8.4 mm) ART (9) Q: 25 EDI [HR]

Follow-Up #19 **02/ago/2019**

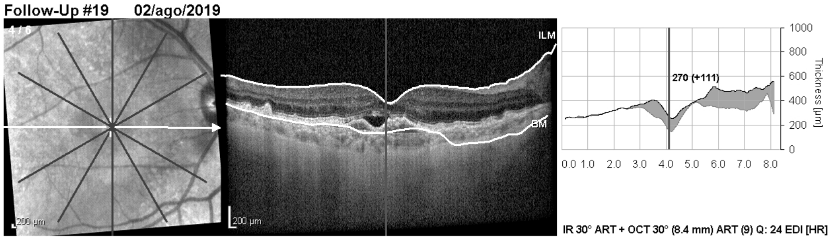

IR 30° ART + OCT 30° (8.4 mm) ART (9) Q: 24 EDI [HR]

Notes:	
Date: 03/01/2024	Signature:

Diagnosis: Comment:

Follow-Up #20 **29/ago/2019**

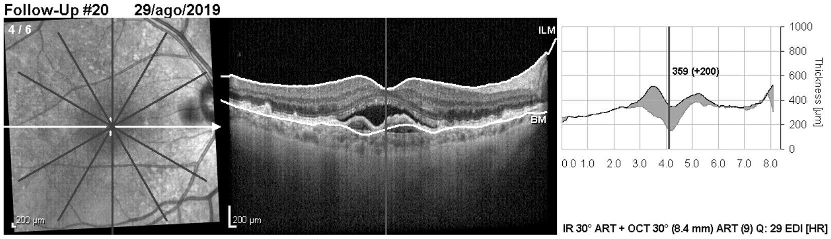

IR 30° ART + OCT 30° (8.4 mm) ART (9) Q: 29 EDI [HR]

Follow-Up #21 **21/out/2019**

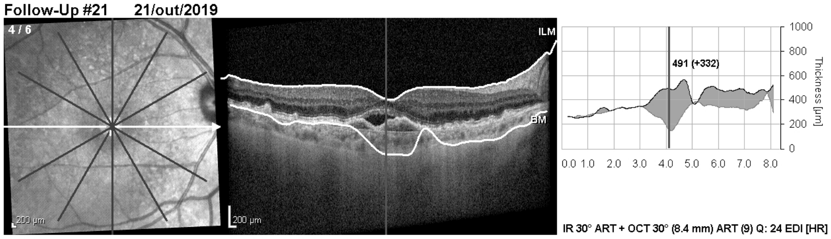

IR 30° ART + OCT 30° (8.4 mm) ART (9) Q: 24 EDI [HR]

Follow-Up #22 **03/dez/2019**

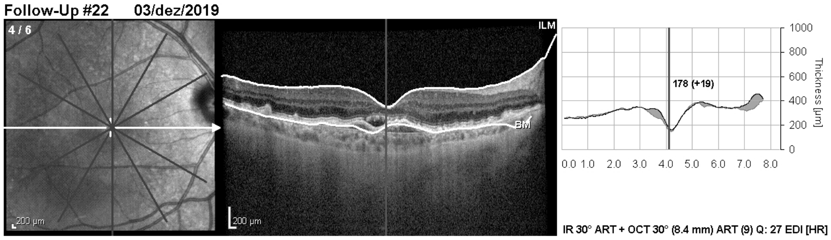

IR 30° ART + OCT 30° (8.4 mm) ART (9) Q: 27 EDI [HR]

Follow-Up #23 **05/mar/2020**

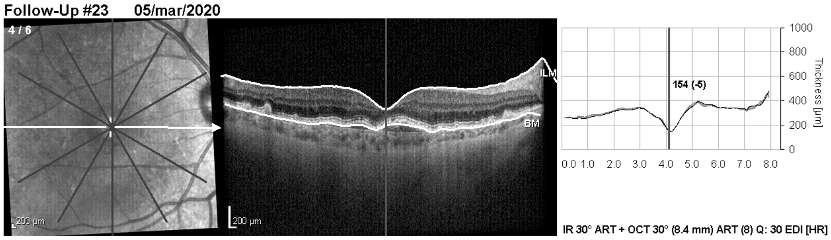

IR 30° ART + OCT 30° (8.4 mm) ART (8) Q: 30 EDI [HR]

Notes:

Date: 03/01/2024 Signature:

[illegible] [illegible]

Follow-Up #24 **27/abr/2020**

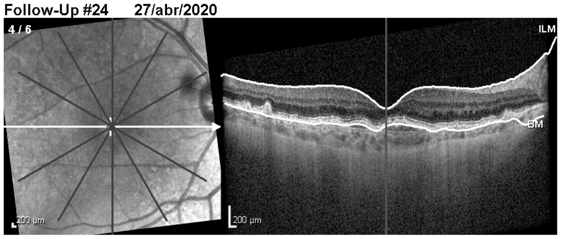

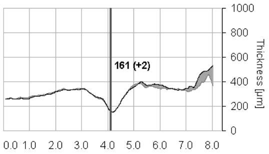

IR 30° ART + OCT 30° (8.4 mm) ART (9) Q: 26 EDI [HR]

Follow-Up #25 **13/jul/2020**

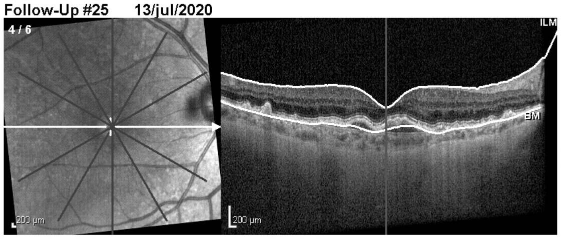

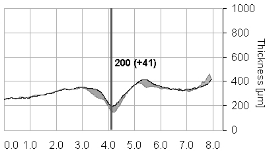

IR 30° ART + OCT 30.0° (8.4 mm) ART (9) Q: 25 EDI [HR]

Follow-Up #26 **26/ago/2020**

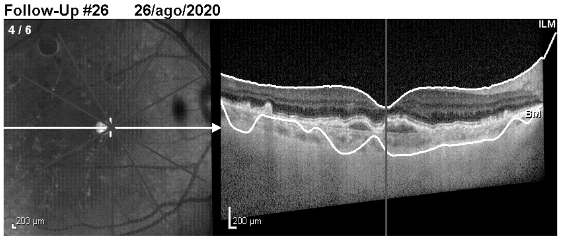

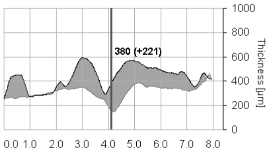

IR 30° ART + OCT 30° (8.4 mm) ART (7) Q: 20 EDI [HR]

Follow-Up #27 **08/out/2020**

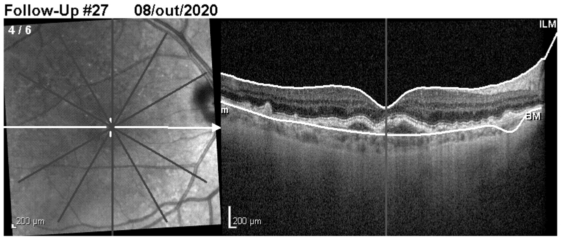

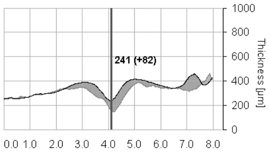

IR 30° ART + OCT 30° (8.4 mm) ART (9) Q: 25 EDI [HR]

Notes:
Date: 03/01/2024 Signature:

Follow-Up #28 29/jan/2021

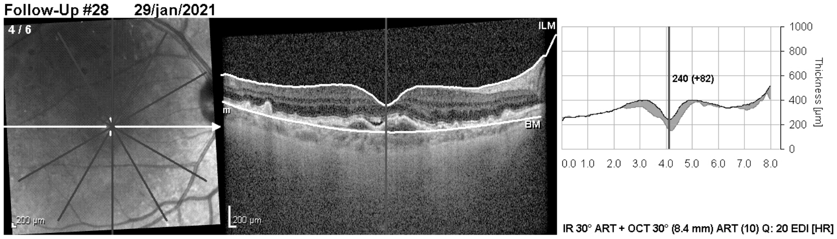

IR 30° ART + OCT 30° (8.4 mm) ART (10) Q: 20 EDI [HR]

Follow-Up #29 01/abr/2021

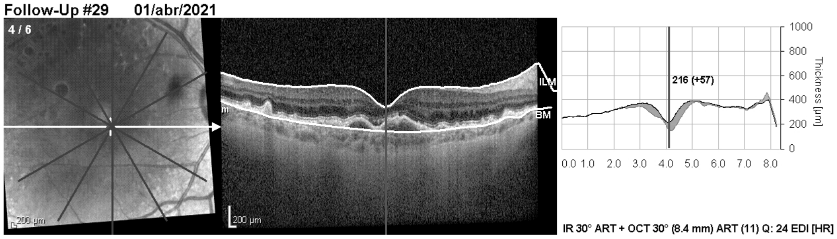

IR 30° ART + OCT 30° (8.4 mm) ART (11) Q: 24 EDI [HR]

Follow-Up #30 20/mai/2021

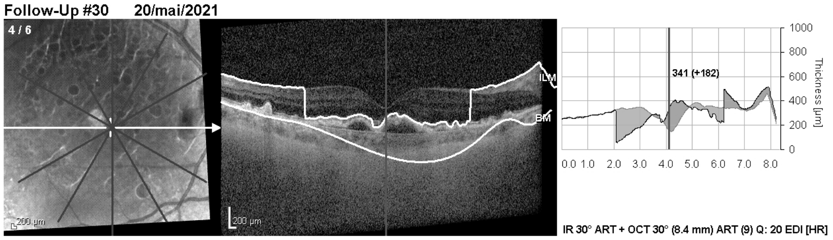

IR 30° ART + OCT 30° (8.4 mm) ART (9) Q: 20 EDI [HR]

Follow-Up #31 24/mai/2021

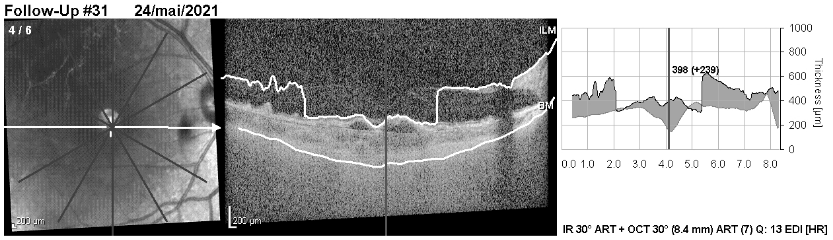

IR 30° ART + OCT 30° (8.4 mm) ART (7) Q: 13 EDI [HR]

Notes:

Date: 03/01/2024 Signature:

Follow-Up #32 **13/ago/2021**

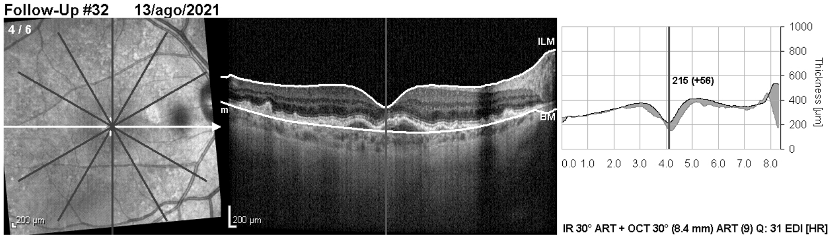

IR 30° ART + OCT 30° (8.4 mm) ART (9) Q: 31 EDI [HR]

Follow-Up #33 **18/out/2021**

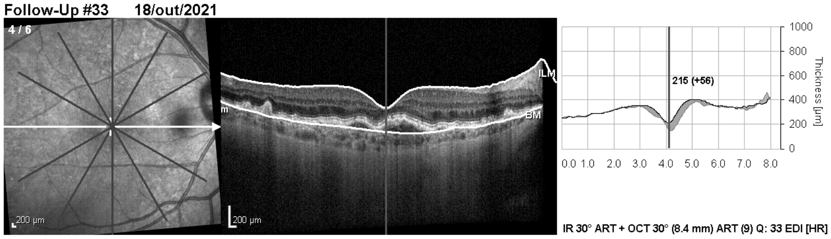

IR 30° ART + OCT 30° (8.4 mm) ART (9) Q: 33 EDI [HR]

Follow-Up #34 **13/jan/2022**

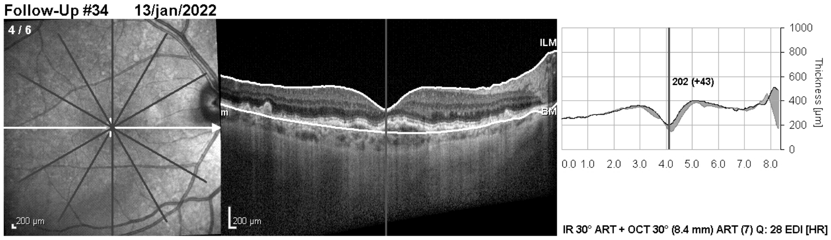

IR 30° ART + OCT 30° (8.4 mm) ART (7) Q: 28 EDI [HR]

Follow-Up #35 **02/mai/2022**

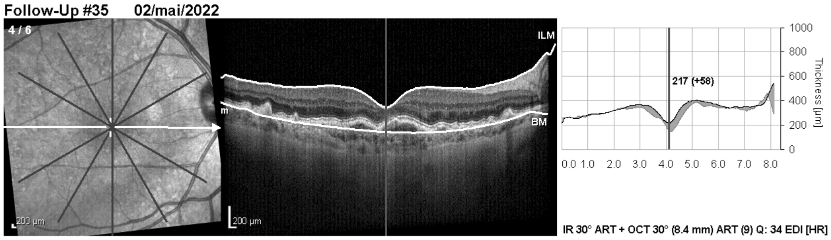

IR 30° ART + OCT 30° (8.4 mm) ART (9) Q: 34 EDI [HR]

Notes:

Date: 03/01/2024 Signature:

Follow-Up #36 01/jul/2022

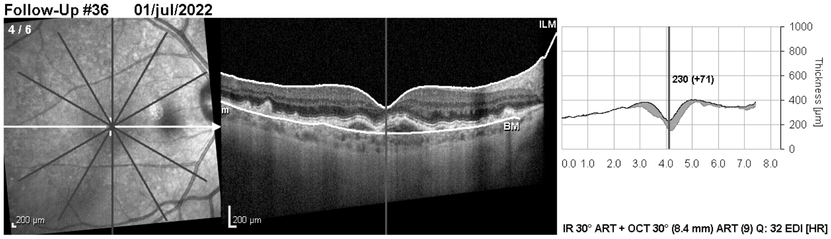

IR 30° ART + OCT 30° (8.4 mm) ART (9) Q: 32 EDI [HR]

Follow-Up #37 26/ago/2022

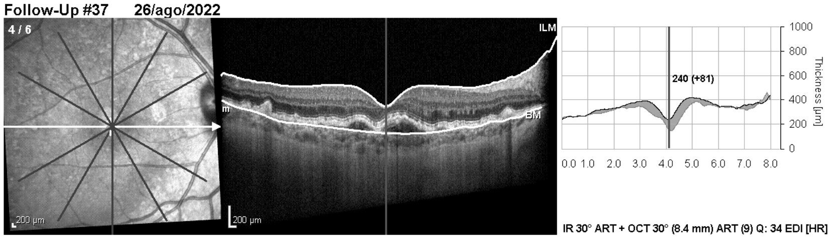

IR 30° ART + OCT 30° (8.4 mm) ART (9) Q: 34 EDI [HR]

Follow-Up #38 12/set/2022

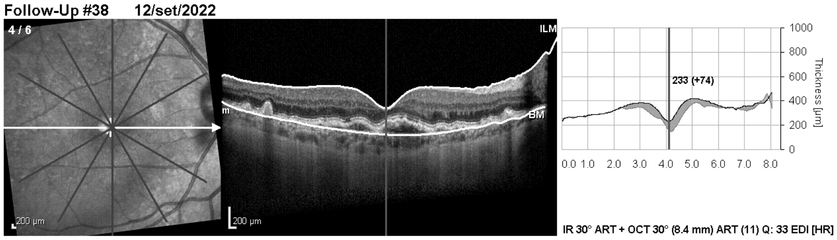

IR 30° ART + OCT 30° (8.4 mm) ART (11) Q: 33 EDI [HR]

Follow-Up #39 24/nov/2022

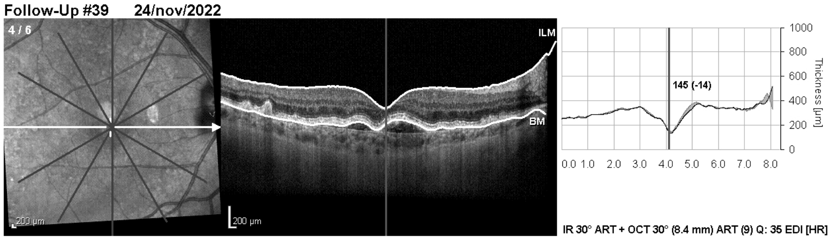

IR 30° ART + OCT 30° (8.4 mm) ART (9) Q: 35 EDI [HR]

Notes:

Date: 03/01/2024 Signature:

[illegible]

Follow-Up #40 **27/fev/2023**

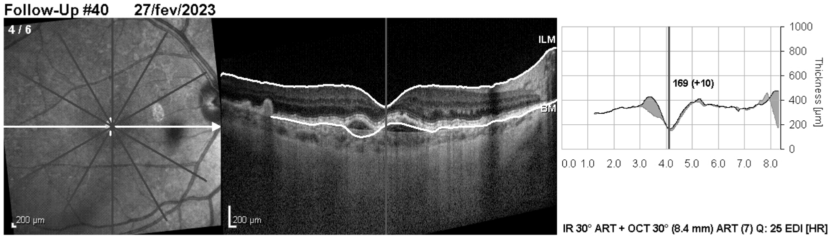

Follow-Up #41 **21/abr/2023**

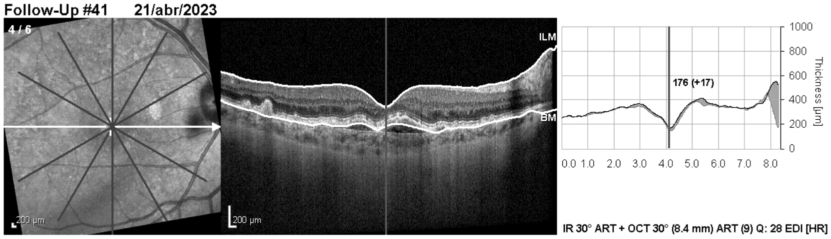

Follow-Up #42 **19/jun/2023**

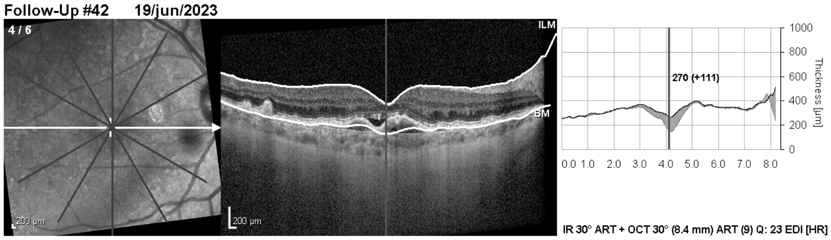

Reference **06/out/2023**

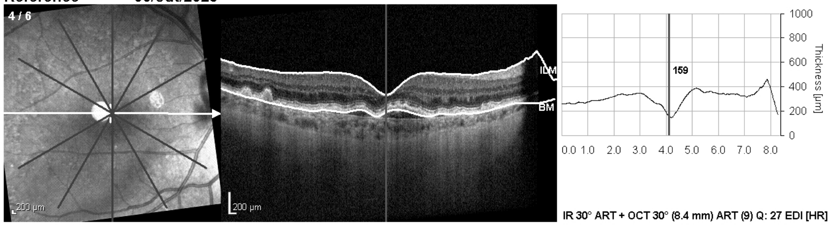

Notes:

Date: 03/01/2024 Signature:

Follow-Up #44 27/nov/2023

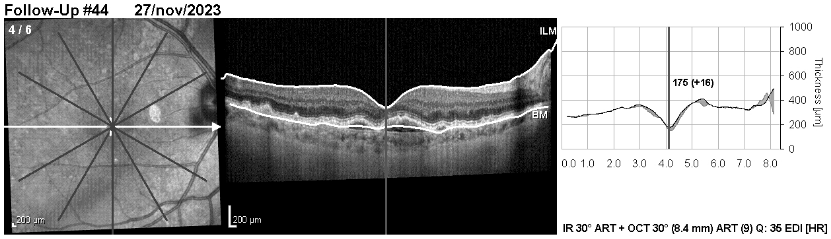

IR 30° ART + OCT 30° (8.4 mm) ART (9) Q: 35 EDI [HR]

Notes:	
Date: 03/01/2024	Signature:

2 Left eye

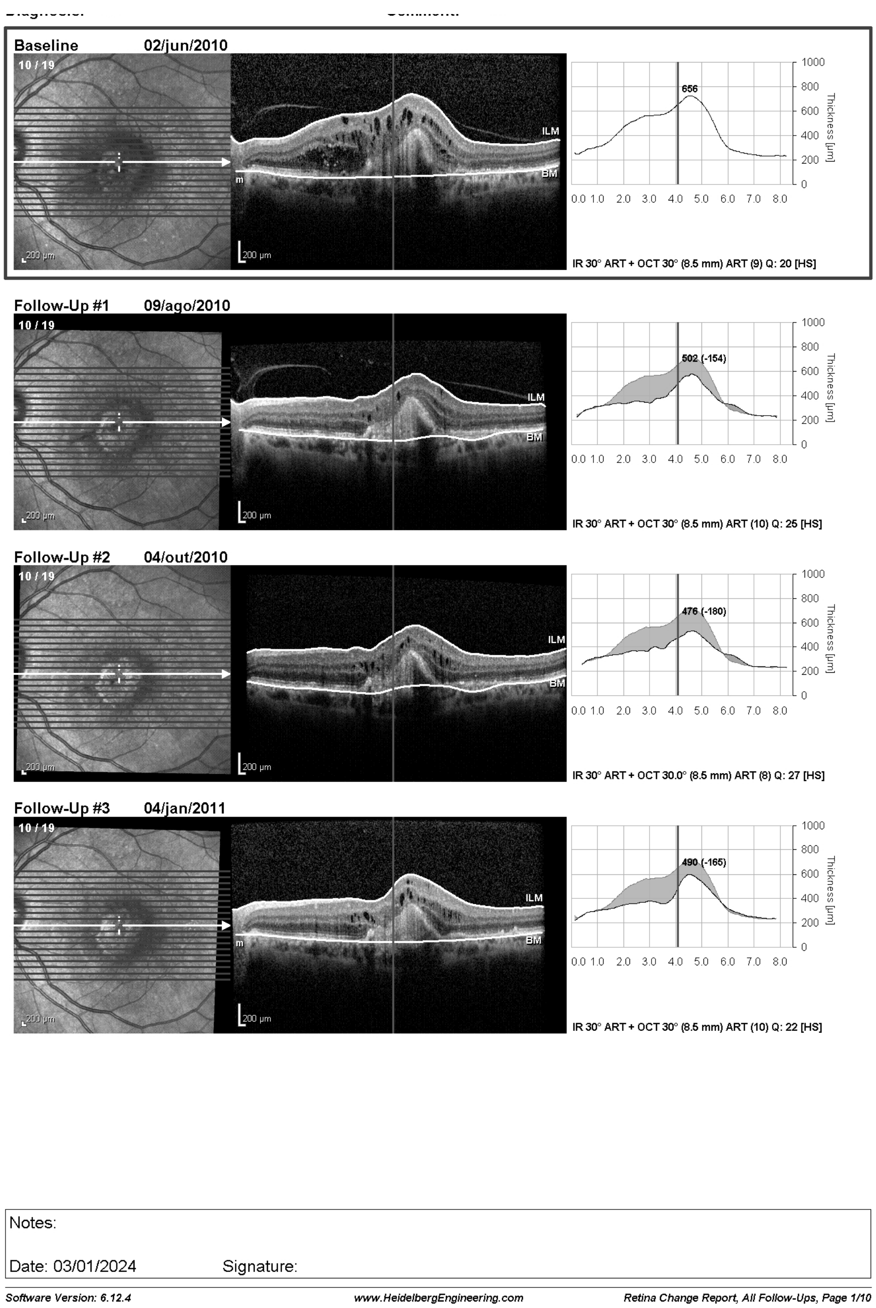
Baseline 02/jun/2010
10 / 19
ILM
BM
656
Thickness [µm]
IR 30° ART + OCT 30° (8.5 mm) ART (9) Q: 20 [HS]
Follow-Up #1 09/ago/2010
502 (-154)
IR 30° ART + OCT 30° (8.5 mm) ART (10) Q: 25 [HS]
Follow-Up #2 04/out/2010
476 (-180)
IR 30° ART + OCT 30.0° (8.5 mm) ART (8) Q: 27 [HS]
Follow-Up #3 04/jan/2011
490 (-165)
IR 30° ART + OCT 30° (8.5 mm) ART (10) Q: 22 [HS]
200 µm
Notes:
Date: 03/01/2024 Signature:
Software Version: 6.12.4
www.HeidelbergEngineering.com
Retina Change Report, All Follow-Ups, Page 1/10

Follow-Up #4 **23/fev/2011**

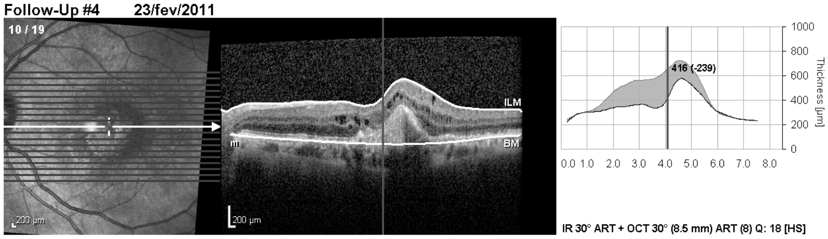

IR 30° ART + OCT 30° (8.5 mm) ART (8) Q: 18 [HS]

Follow-Up #5 **08/abr/2011**

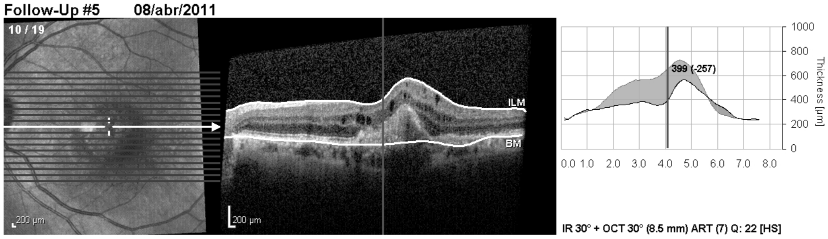

IR 30° + OCT 30° (8.5 mm) ART (7) Q: 22 [HS]

Follow-Up #6 **17/mai/2011**

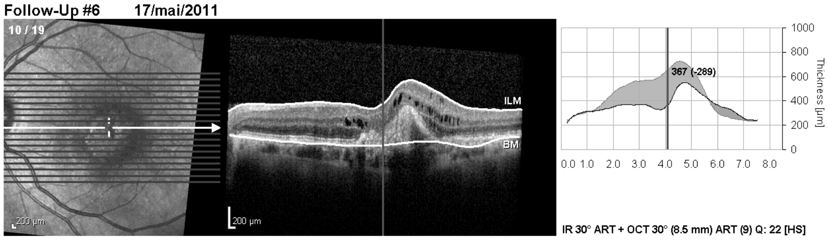

IR 30° ART + OCT 30° (8.5 mm) ART (9) Q: 22 [HS]

Follow-Up #7 **31/mai/2011**

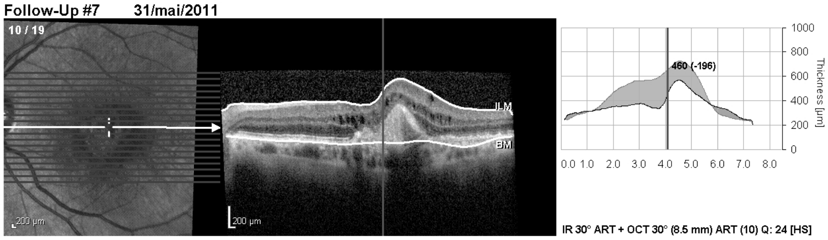

IR 30° ART + OCT 30° (8.5 mm) ART (10) Q: 24 [HS]

Notes:

Date: 03/01/2024 Signature:

[illegible] [illegible]

Follow-Up #8 **05/jul/2011**

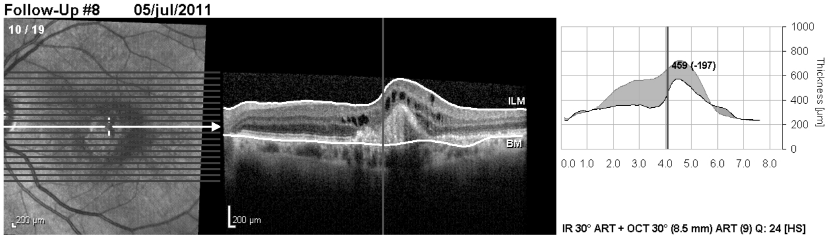

IR 30° ART + OCT 30° (8.5 mm) ART (9) Q: 24 [HS]

Follow-Up #9 **22/ago/2011**

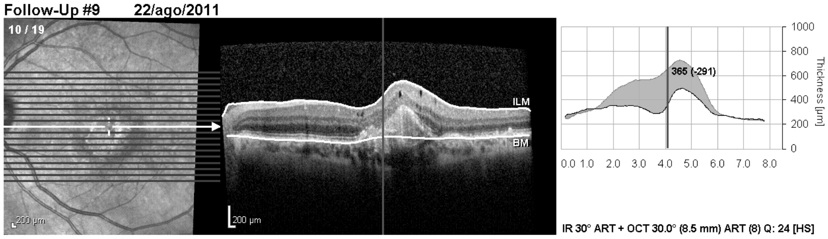

IR 30° ART + OCT 30.0° (8.5 mm) ART (8) Q: 24 [HS]

Follow-Up #10 **12/set/2011**

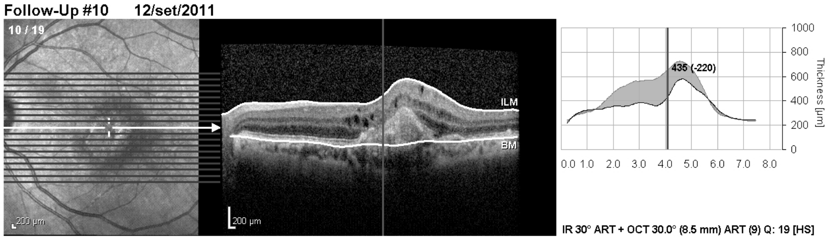

IR 30° ART + OCT 30.0° (8.5 mm) ART (9) Q: 19 [HS]

Follow-Up #11 **27/set/2011**

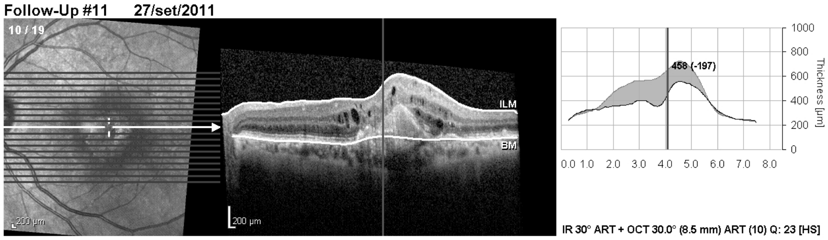

IR 30° ART + OCT 30.0° (8.5 mm) ART (10) Q: 23 [HS]

Notes:

Date: 03/01/2024 Signature:

Follow-Up #12 20/out/2011

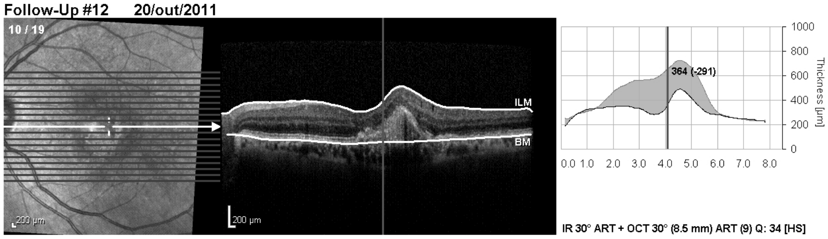

IR 30° ART + OCT 30° (8.5 mm) ART (9) Q: 34 [HS]

Follow-Up #13 22/nov/2011

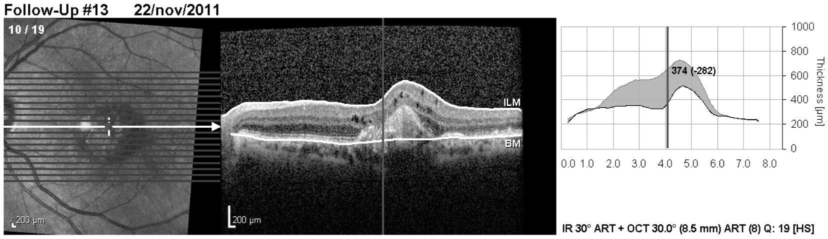

IR 30° ART + OCT 30.0° (8.5 mm) ART (8) Q: 19 [HS]

Follow-Up #14 20/dez/2011

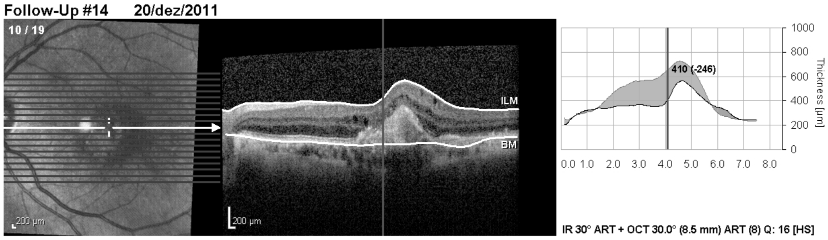

IR 30° ART + OCT 30.0° (8.5 mm) ART (8) Q: 16 [HS]

Follow-Up #15 26/dez/2011

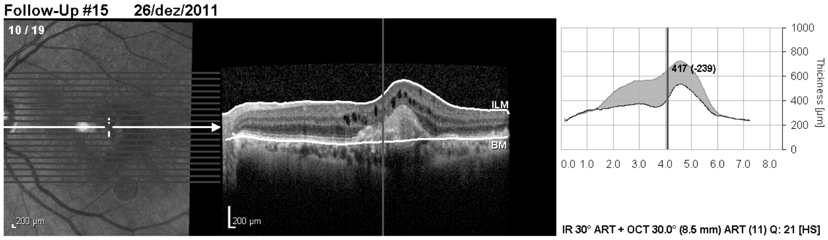

IR 30° ART + OCT 30.0° (8.5 mm) ART (11) Q: 21 [HS]

Notes:

Date: 03/01/2024 Signature:

[illegible]

Follow-Up #16   23/jan/2012

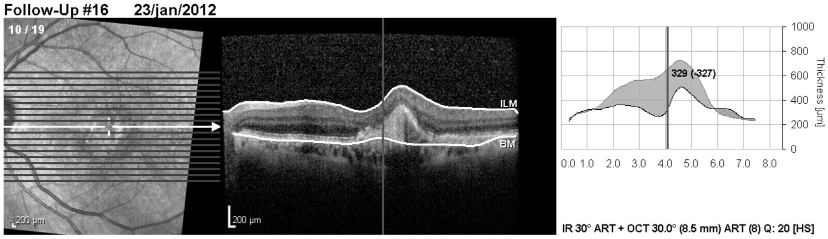

IR 30° ART + OCT 30.0° (8.5 mm) ART (8) Q: 20 [HS]

Follow-Up #17   14/fev/2012

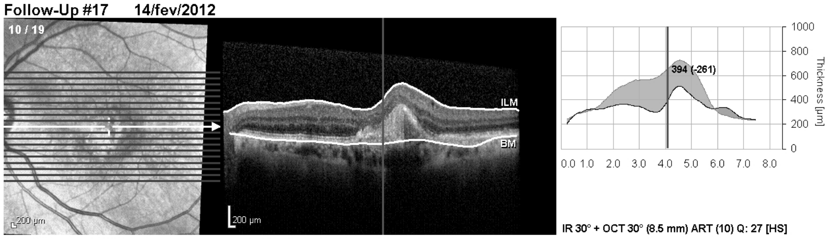

IR 30° + OCT 30° (8.5 mm) ART (10) Q: 27 [HS]

Follow-Up #18   16/mar/2012

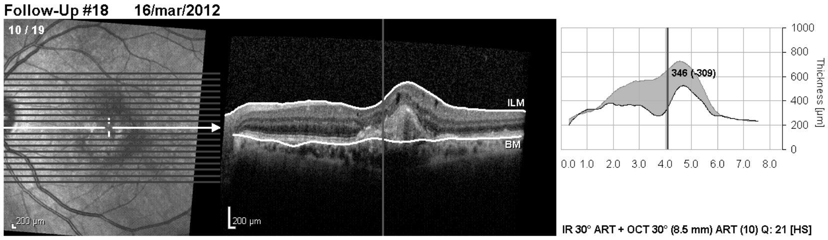

IR 30° ART + OCT 30° (8.5 mm) ART (10) Q: 21 [HS]

Follow-Up #19   17/abr/2012

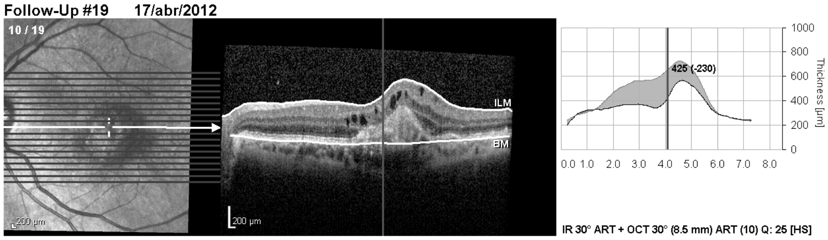

IR 30° ART + OCT 30° (8.5 mm) ART (10) Q: 25 [HS]

Notes:

Date: 03/01/2024   Signature:

Follow-Up #20 25/mai/2012

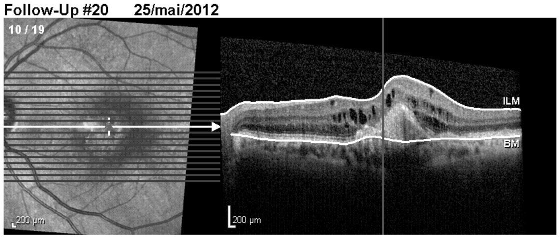

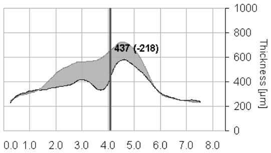

IR 30° ART + OCT 30° (8.5 mm) ART (8) Q: 25 [HS]

Follow-Up #21 12/jun/2012

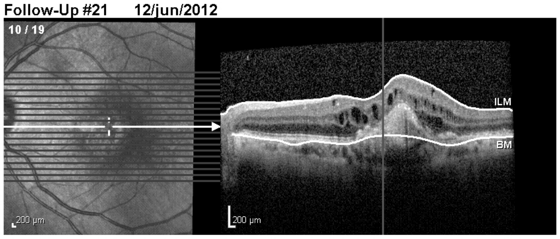

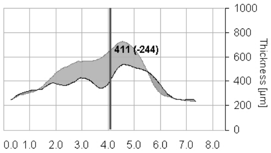

IR 30° ART + OCT 30.0° (8.5 mm) ART (9) Q: 19 [HS]

Follow-Up #22 13/jul/2012

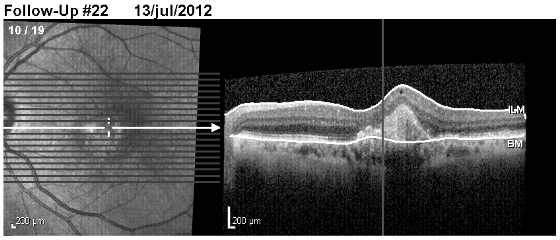

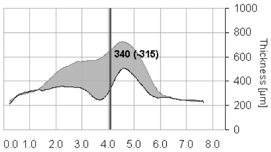

IR 30° ART + OCT 30° (8.5 mm) ART (8) Q: 22 [HS]

Follow-Up #23 31/ago/2012

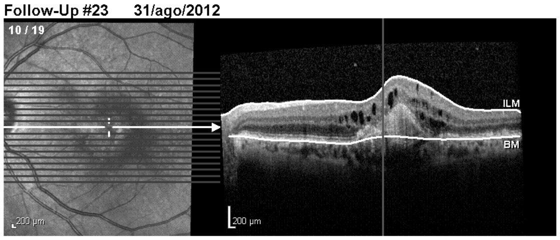

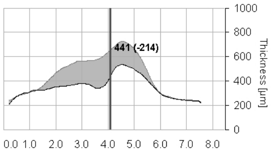

IR 30° ART + OCT 30.0° (8.5 mm) ART (9) Q: 28 [HS]

Notes:

Date: 03/01/2024 Signature:

Follow-Up #24 24/set/2012

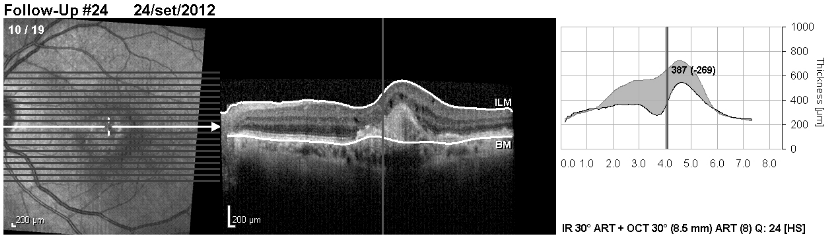

IR 30° ART + OCT 30° (8.5 mm) ART (8) Q: 24 [HS]

Follow-Up #25 19/out/2012

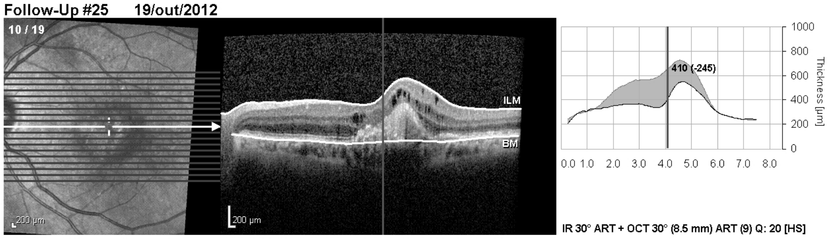

IR 30° ART + OCT 30° (8.5 mm) ART (9) Q: 20 [HS]

Follow-Up #26 02/nov/2012

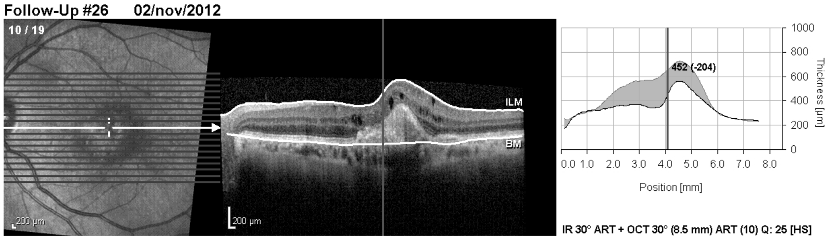

IR 30° ART + OCT 30° (8.5 mm) ART (10) Q: 25 [HS]

Follow-Up #27 11/dez/2012

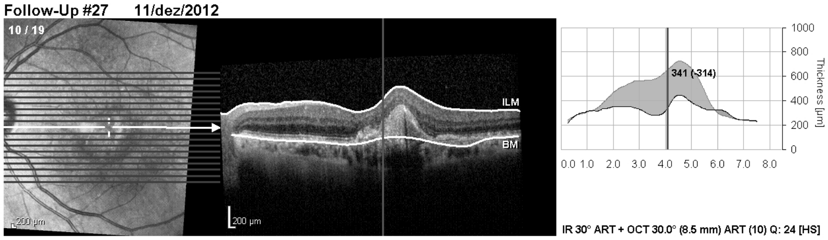

IR 30° ART + OCT 30.0° (8.5 mm) ART (10) Q: 24 [HS]

Notes:

Date: 03/01/2024 Signature:

Follow-Up #28 14/jan/2013

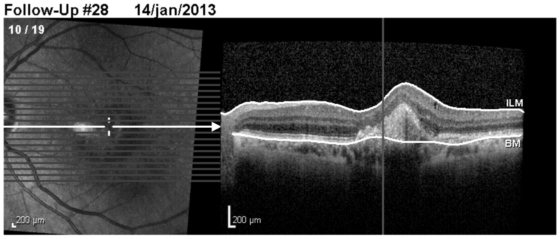

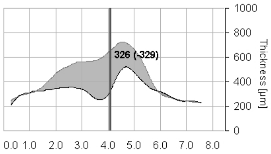

IR 30° ART + OCT 30.0° (8.5 mm) ART (8) Q: 23 [HS]

Follow-Up #29 29/jan/2013

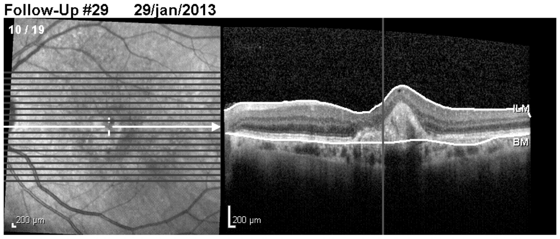

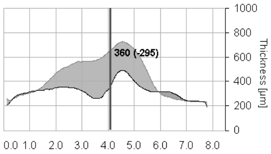

IR 30° ART + OCT 30.0° (8.5 mm) ART (10) Q: 25 [HS]

Follow-Up #30 11/mar/2013

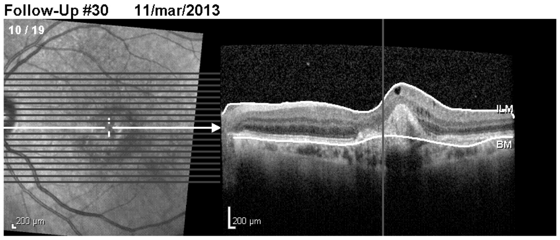

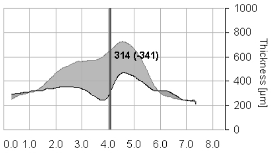

IR 30° ART + OCT 30° (8.5 mm) ART (8) Q: 22 [HS]

Follow-Up #31 12/abr/2013

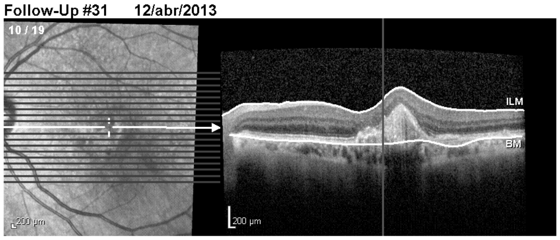

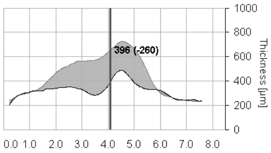

IR 30° ART + OCT 30.0° (8.5 mm) ART (8) Q: 21 [HS]

Notes:

Date: 03/01/2024 Signature:

Follow-Up #32 13/mai/2013

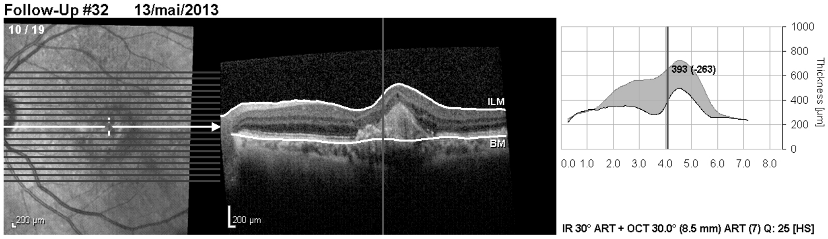

Follow-Up #33 25/jun/2013

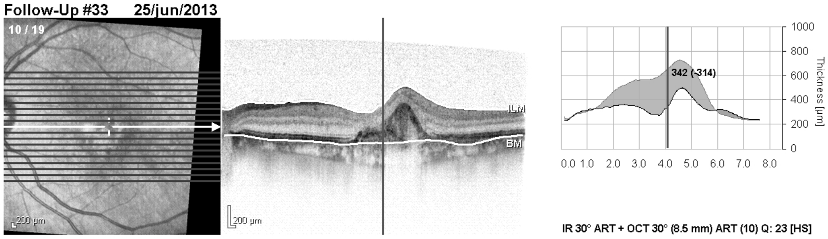

Follow-Up #34 26/ago/2013

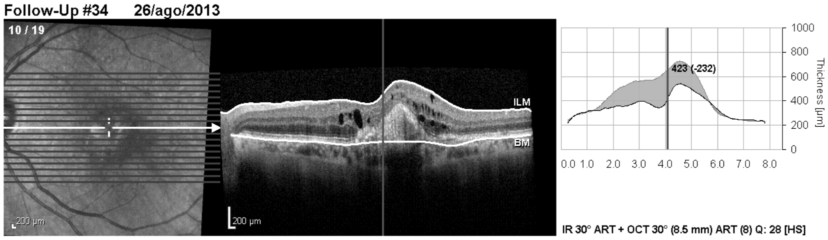

Follow-Up #35 02/set/2013

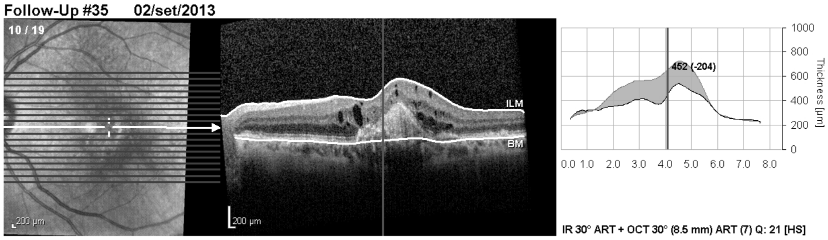

Notes:

Date: 03/01/2024 Signature:

Follow-Up #36 01/out/2013

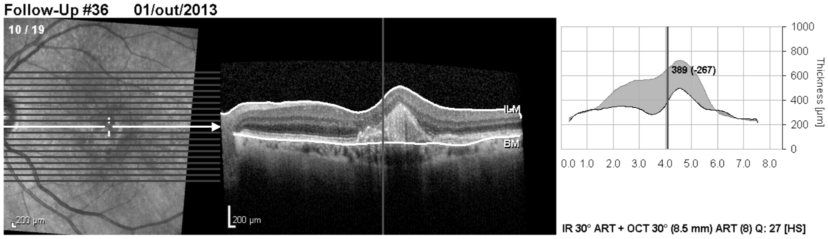

Follow-Up #37 04/nov/2013

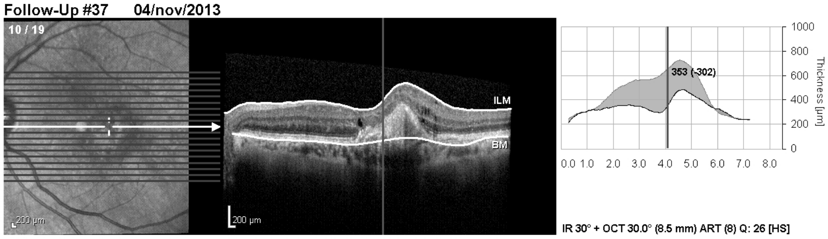

Follow-Up #38 10/jan/2014

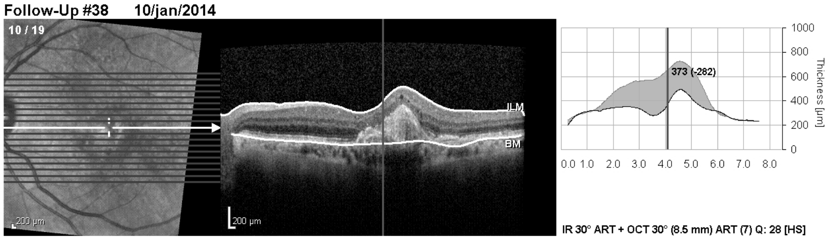

Follow-Up #39 21/fev/2014

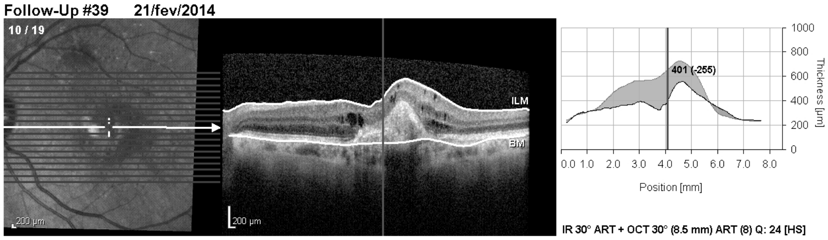

Notes:

Date: 03/01/2024 Signature:

[illegible]

Baseline **10/jan/2014**

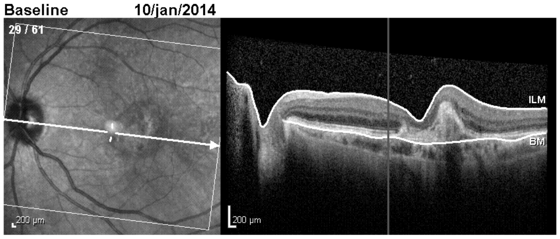

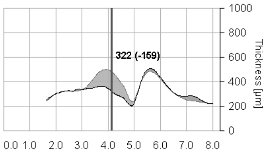

IR 30° ART + OCT 30.0° (8.3 mm) ART (7) Q: 28 [HS]

Follow-Up #1 **21/fev/2014**

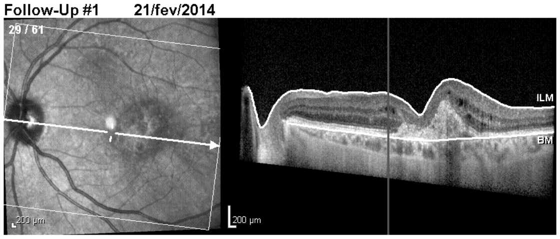

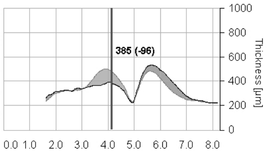

IR 30° + OCT 30° (8.3 mm) ART (9) Q: 26 EDI [HS]

Follow-Up #2 **19/mar/2014**

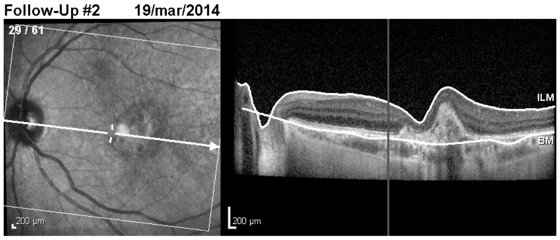

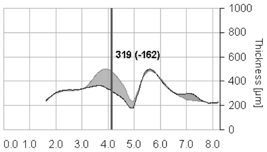

IR 30° ART + OCT 30.0° (8.3 mm) ART (8) Q: 23 EDI [HS]

Follow-Up #3 **22/abr/2014**

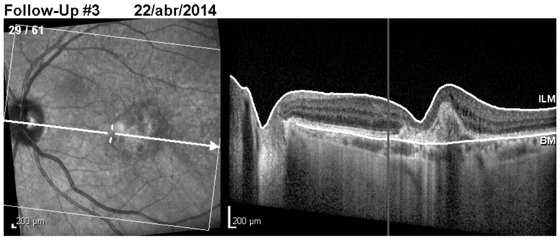

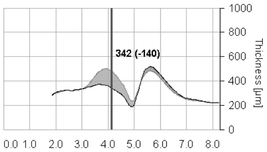

IR 30° ART + OCT 30.0° (8.3 mm) ART (9) Q: 24 EDI [HS]

Notes:

Date: 03/01/2024 Signature:

Follow-Up #4 **17/jun/2014**

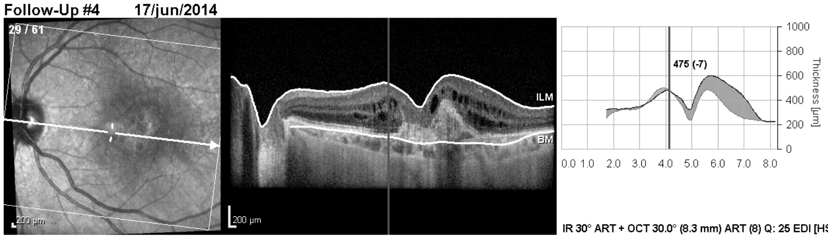

IR 30° ART + OCT 30.0° (8.3 mm) ART (8) Q: 25 EDI [HS]

Follow-Up #5 **02/jul/2014**

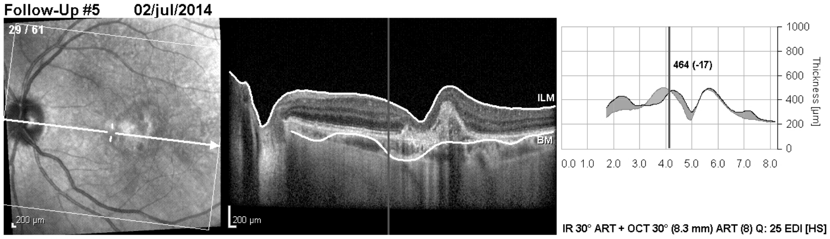

IR 30° ART + OCT 30° (8.3 mm) ART (8) Q: 25 EDI [HS]

Follow-Up #6 **23/set/2014**

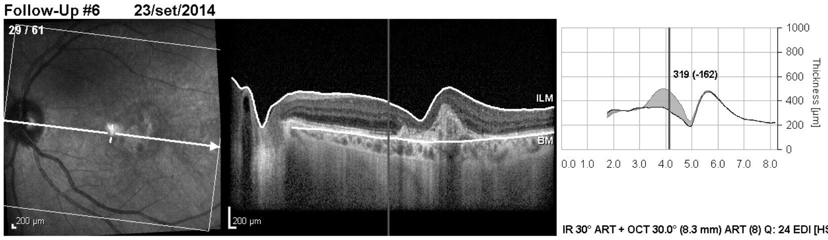

IR 30° ART + OCT 30.0° (8.3 mm) ART (8) Q: 24 EDI [HS]

Follow-Up #7 **11/nov/2014**

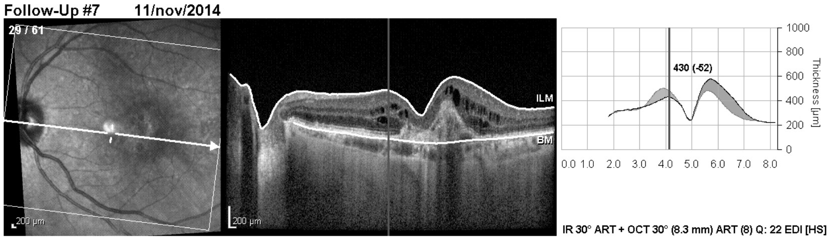

IR 30° ART + OCT 30° (8.3 mm) ART (8) Q: 22 EDI [HS]

Notes:

Date: 03/01/2024 Signature:

[illegible]

Follow-Up #8 **15/dez/2014**

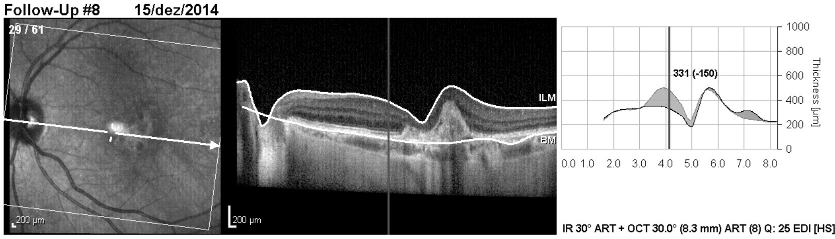

IR 30° ART + OCT 30.0° (8.3 mm) ART (8) Q: 25 EDI [HS]

Follow-Up #9 **26/jan/2015**

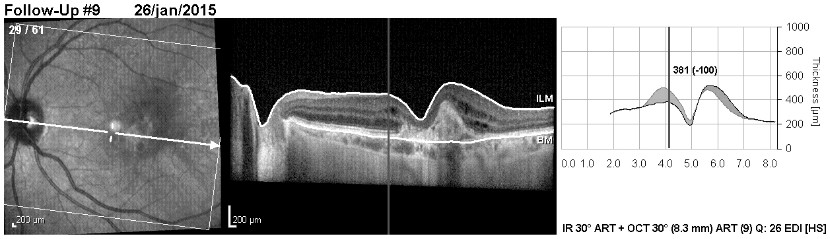

IR 30° ART + OCT 30° (8.3 mm) ART (9) Q: 26 EDI [HS]

Follow-Up #10 **20/fev/2015**

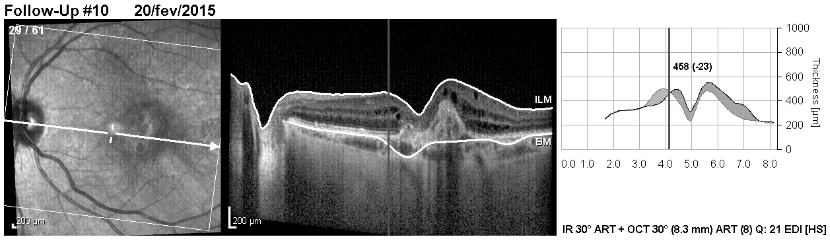

IR 30° ART + OCT 30° (8.3 mm) ART (8) Q: 21 EDI [HS]

Follow-Up #11 **24/mar/2015**

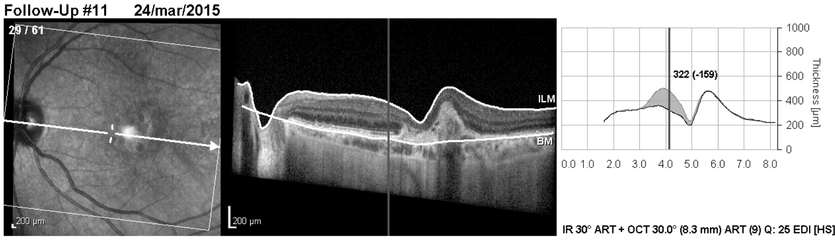

IR 30° ART + OCT 30.0° (8.3 mm) ART (9) Q: 25 EDI [HS]

Notes:

Date: 03/01/2024 Signature:

Follow-Up #12 11/mai/2015

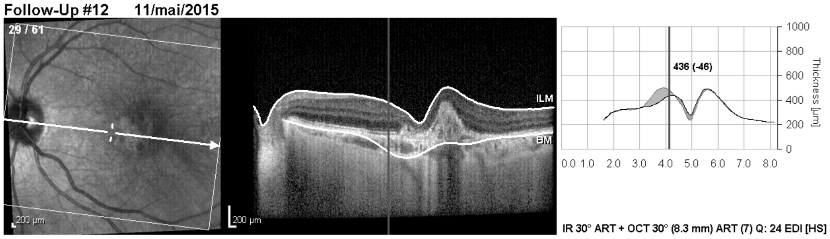

IR 30° ART + OCT 30° (8.3 mm) ART (7) Q: 24 EDI [HS]

Follow-Up #13 03/jun/2015

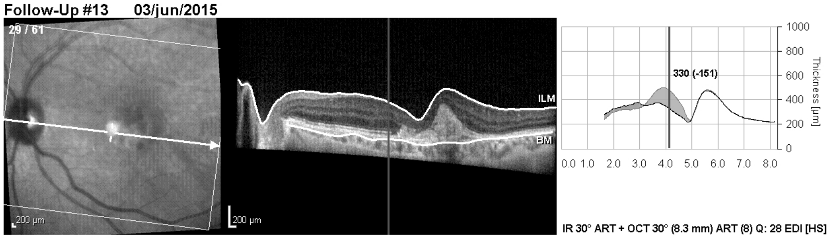

IR 30° ART + OCT 30° (8.3 mm) ART (8) Q: 28 EDI [HS]

Follow-Up #14 01/jul/2015

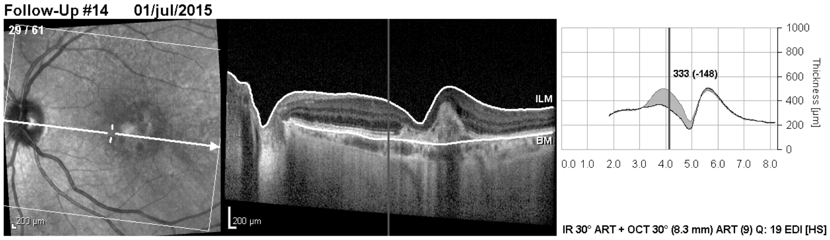

IR 30° ART + OCT 30° (8.3 mm) ART (9) Q: 19 EDI [HS]

Follow-Up #15 05/ago/2015

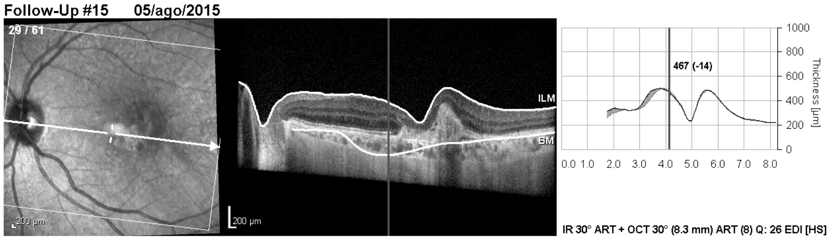

IR 30° ART + OCT 30° (8.3 mm) ART (8) Q: 26 EDI [HS]

Notes:

Date: 03/01/2024 Signature:

Follow-Up #16 29/set/2015

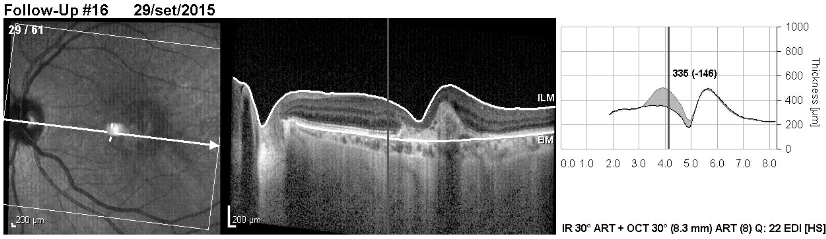

IR 30° ART + OCT 30° (8.3 mm) ART (8) Q: 22 EDI [HS]

Follow-Up #17 09/dez/2015

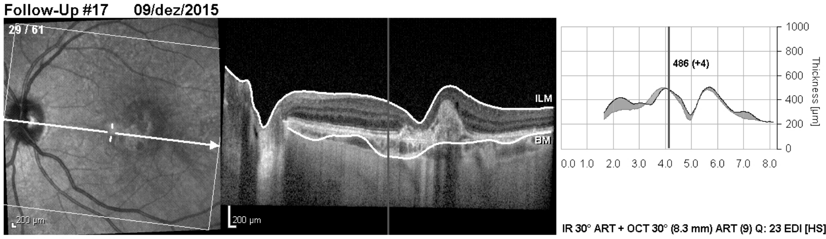

IR 30° ART + OCT 30° (8.3 mm) ART (9) Q: 23 EDI [HS]

Follow-Up #18 14/jan/2016

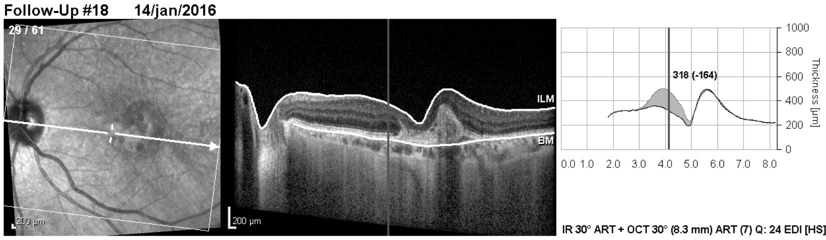

IR 30° ART + OCT 30° (8.3 mm) ART (7) Q: 24 EDI [HS]

Follow-Up #19 09/mar/2016

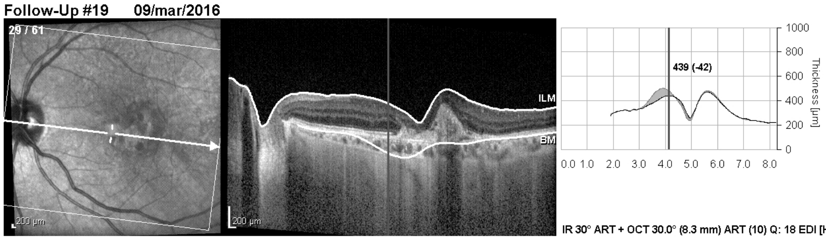

IR 30° ART + OCT 30.0° (8.3 mm) ART (10) Q: 18 EDI [HS]

Notes:

Date: 03/01/2024 Signature:

Follow-Up #20   04/mai/2016

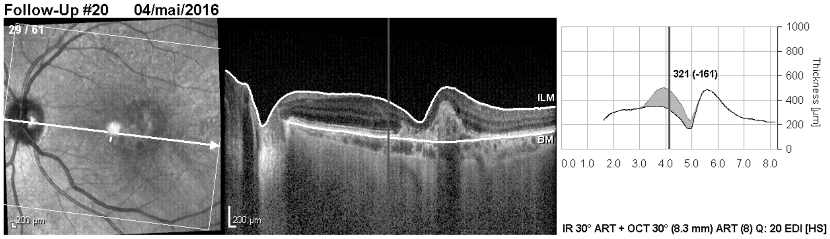

IR 30° ART + OCT 30° (8.3 mm) ART (8) Q: 20 EDI [HS]

Follow-Up #21   05/ago/2016

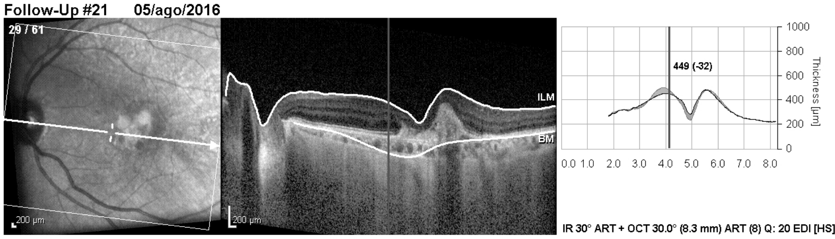

IR 30° ART + OCT 30.0° (8.3 mm) ART (8) Q: 20 EDI [HS]

Follow-Up #22   17/out/2016

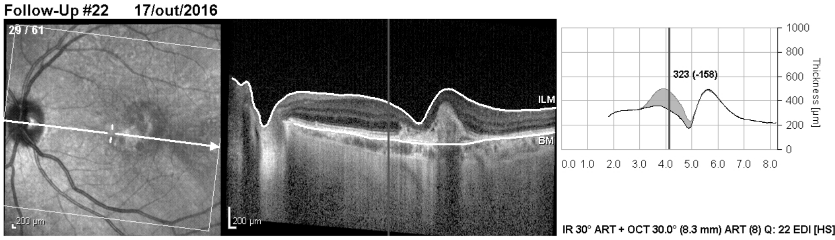

IR 30° ART + OCT 30.0° (8.3 mm) ART (8) Q: 22 EDI [HS]

Follow-Up #23   28/nov/2016

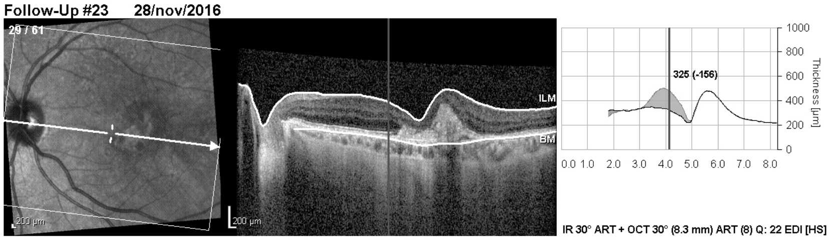

IR 30° ART + OCT 30° (8.3 mm) ART (8) Q: 22 EDI [HS]

Notes:

Date: 03/01/2024   Signature:

Follow-Up #24 27/fev/2017

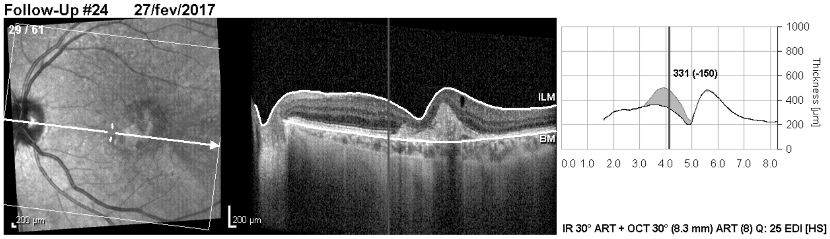

IR 30° ART + OCT 30° (8.3 mm) ART (8) Q: 25 EDI [HS]

Follow-Up #25 05/mai/2017

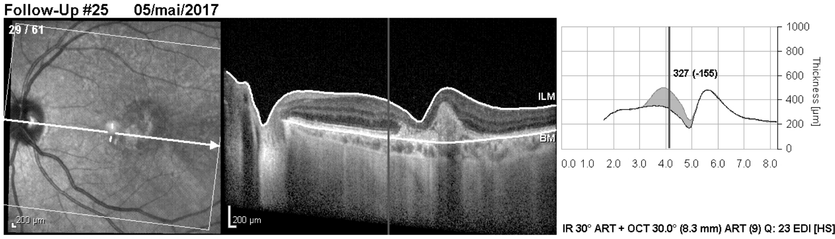

IR 30° ART + OCT 30.0° (8.3 mm) ART (9) Q: 23 EDI [HS]

Follow-Up #26 14/jul/2017

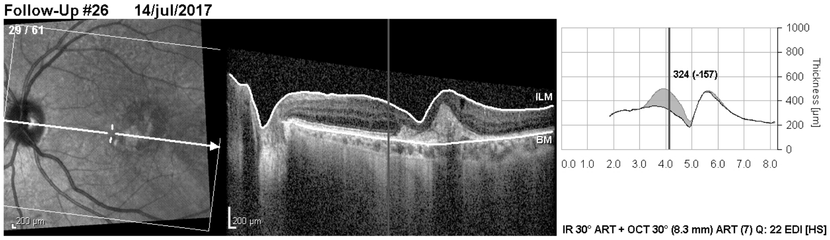

IR 30° ART + OCT 30° (8.3 mm) ART (7) Q: 22 EDI [HS]

Follow-Up #27 05/set/2017

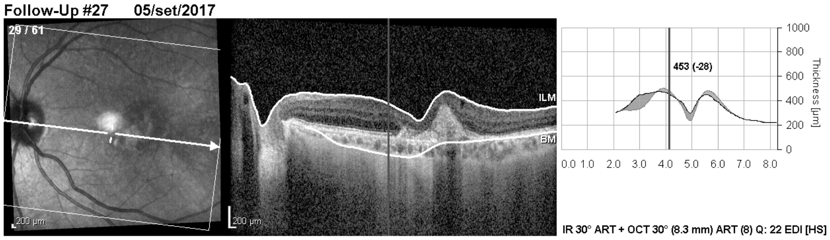

IR 30° ART + OCT 30° (8.3 mm) ART (8) Q: 22 EDI [HS]

Notes:

Date: 03/01/2024 Signature:

[illegible]

Baseline **09/mar/2016**

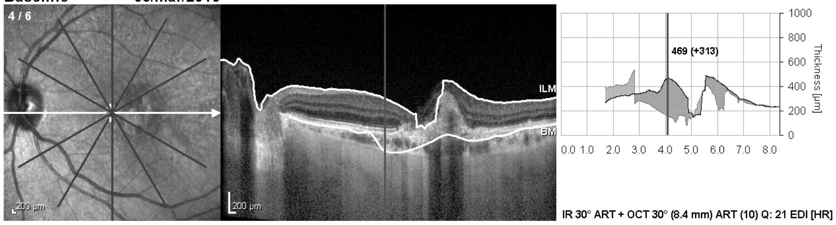

IR 30° ART + OCT 30° (8.4 mm) ART (10) Q: 21 EDI [HR]

Follow-Up #1 **04/mai/2016**

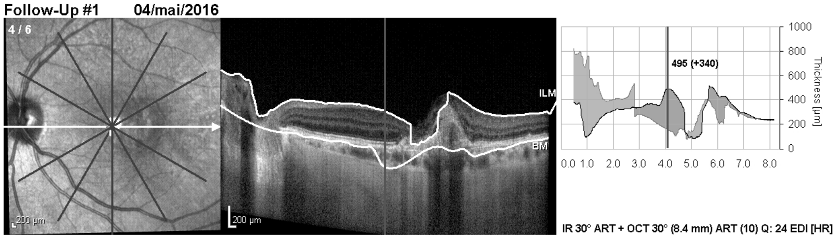

IR 30° ART + OCT 30° (8.4 mm) ART (10) Q: 24 EDI [HR]

Follow-Up #2 **05/ago/2016**

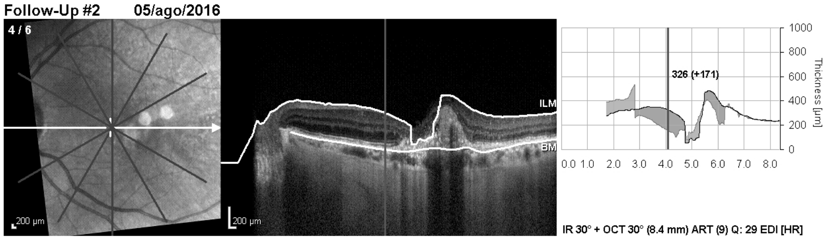

IR 30° + OCT 30° (8.4 mm) ART (9) Q: 29 EDI [HR]

Follow-Up #3 **17/out/2016**

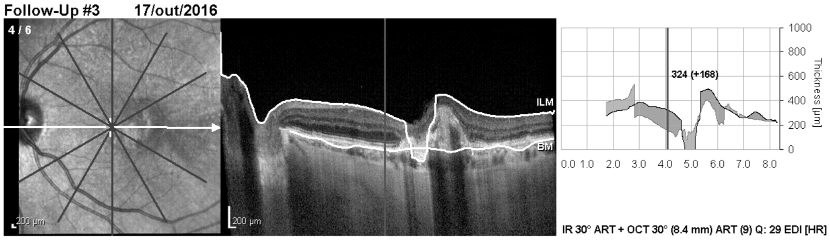

IR 30° ART + OCT 30° (8.4 mm) ART (9) Q: 29 EDI [HR]

Notes:	
Date: 03/01/2024	Signature:

Software Version: 6.12.4 *www.HeidelbergEngineering.com* *Retina Change Report, All Follow-Ups, Page 1/8*

Follow-Up #4 **28/nov/2016**

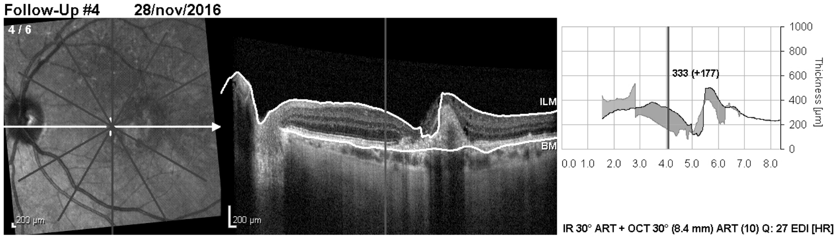

IR 30° ART + OCT 30° (8.4 mm) ART (10) Q: 27 EDI [HR]

Follow-Up #5 **27/fev/2017**

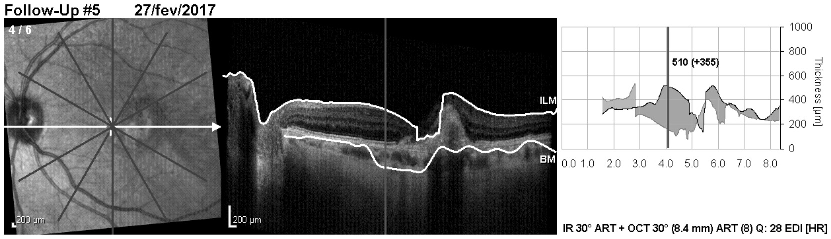

IR 30° ART + OCT 30° (8.4 mm) ART (8) Q: 28 EDI [HR]

Follow-Up #6 **05/mai/2017**

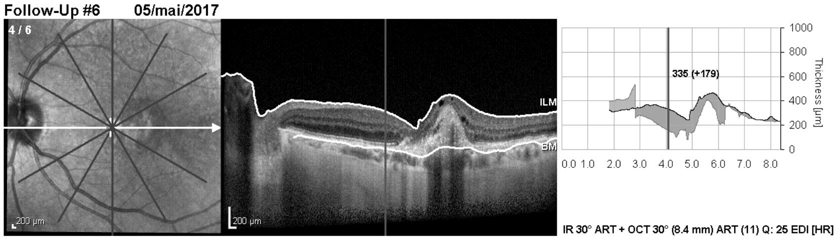

IR 30° ART + OCT 30° (8.4 mm) ART (11) Q: 25 EDI [HR]

Follow-Up #7 **14/jul/2017**

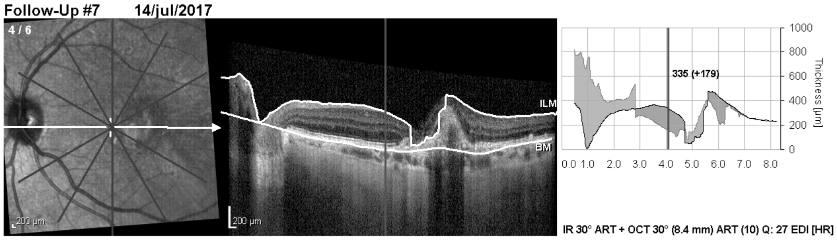

IR 30° ART + OCT 30° (8.4 mm) ART (10) Q: 27 EDI [HR]

Notes:

Date: 03/01/2024 Signature:

Follow-Up #8 **05/set/2017**

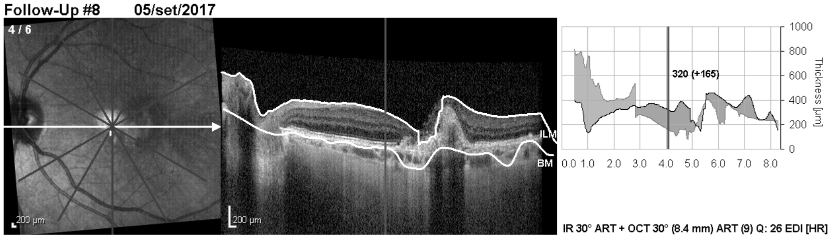

IR 30° ART + OCT 30° (8.4 mm) ART (9) Q: 26 EDI [HR]

Follow-Up #9 **27/nov/2017**

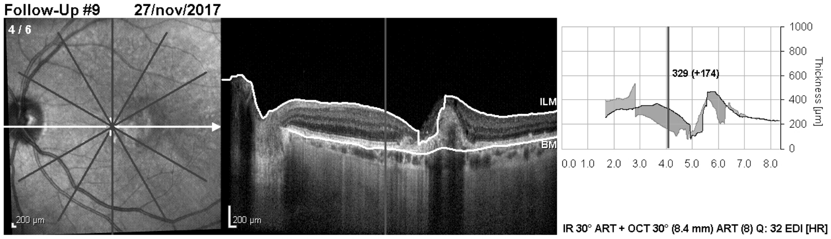

IR 30° ART + OCT 30° (8.4 mm) ART (8) Q: 32 EDI [HR]

Follow-Up #10 **16/jan/2018**

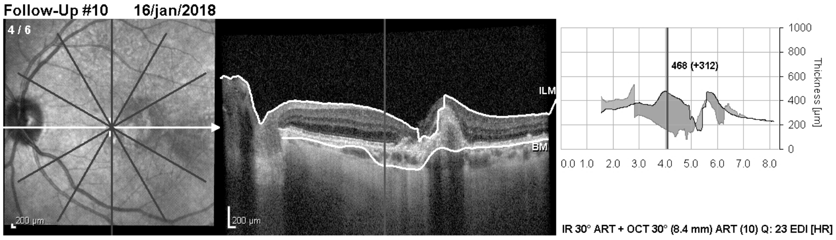

IR 30° ART + OCT 30° (8.4 mm) ART (10) Q: 23 EDI [HR]

Follow-Up #11 **08/mar/2018**

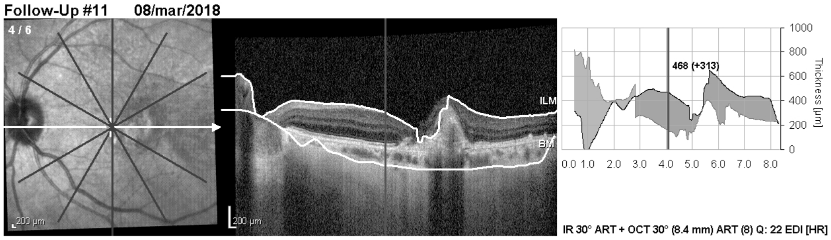

IR 30° ART + OCT 30° (8.4 mm) ART (8) Q: 22 EDI [HR]

Notes:

Date: 03/01/2024 Signature:

[illegible]

Follow-Up #12 02/abr/2018

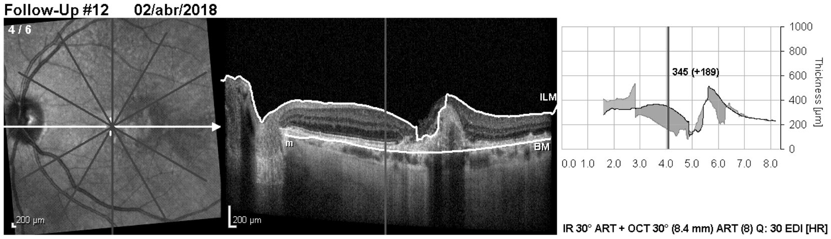

IR 30° ART + OCT 30° (8.4 mm) ART (8) Q: 30 EDI [HR]

Follow-Up #13 05/jun/2018

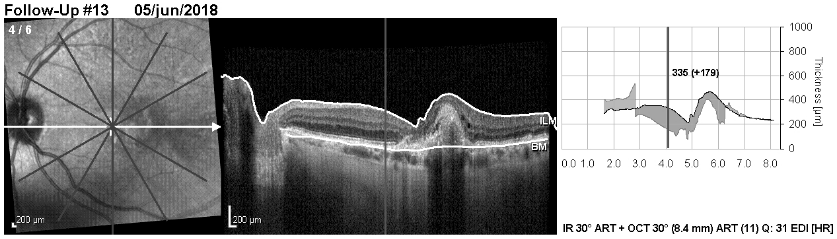

IR 30° ART + OCT 30° (8.4 mm) ART (11) Q: 31 EDI [HR]

Follow-Up #14 13/ago/2018

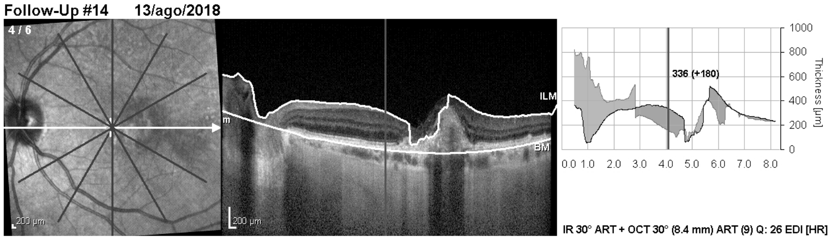

IR 30° ART + OCT 30° (8.4 mm) ART (9) Q: 26 EDI [HR]

Follow-Up #15 20/nov/2018

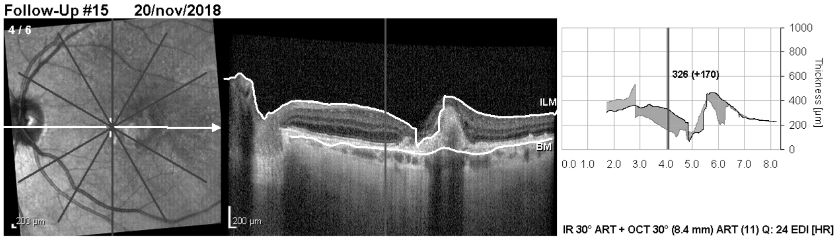

IR 30° ART + OCT 30° (8.4 mm) ART (11) Q: 24 EDI [HR]

Notes:	
Date: 03/01/2024	Signature:

Follow-Up #16   04/fev/2019

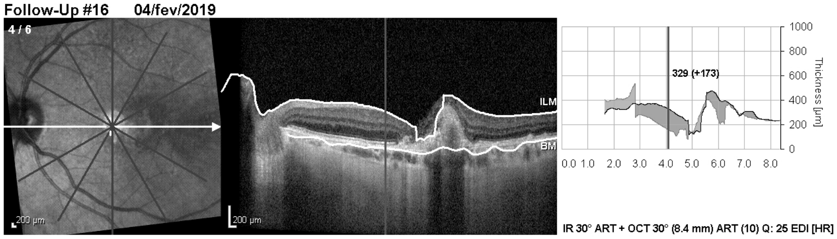

IR 30° ART + OCT 30° (8.4 mm) ART (10) Q: 25 EDI [HR]

Follow-Up #17   15/abr/2019

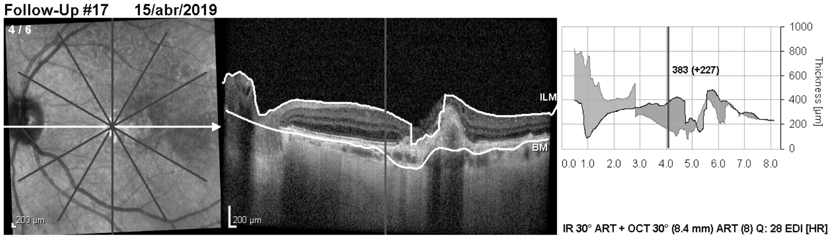

IR 30° ART + OCT 30° (8.4 mm) ART (8) Q: 28 EDI [HR]

Follow-Up #18   15/mai/2019

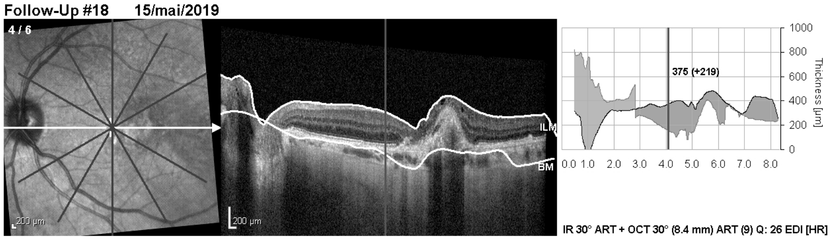

IR 30° ART + OCT 30° (8.4 mm) ART (9) Q: 26 EDI [HR]

Follow-Up #19   02/ago/2019

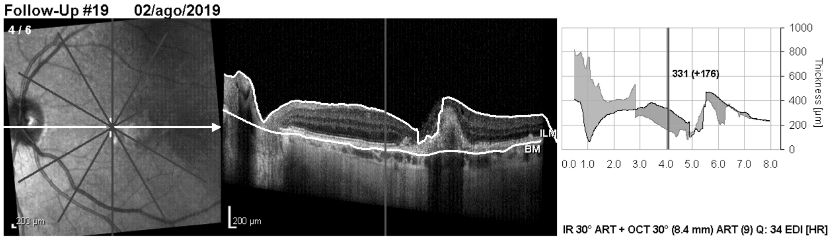

IR 30° ART + OCT 30° (8.4 mm) ART (9) Q: 34 EDI [HR]

Notes:

Date: 03/01/2024   Signature:

[illegible]

Follow-Up #20 29/ago/2019

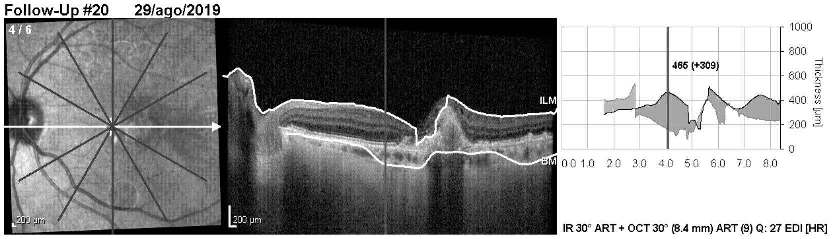

Follow-Up #21 21/out/2019

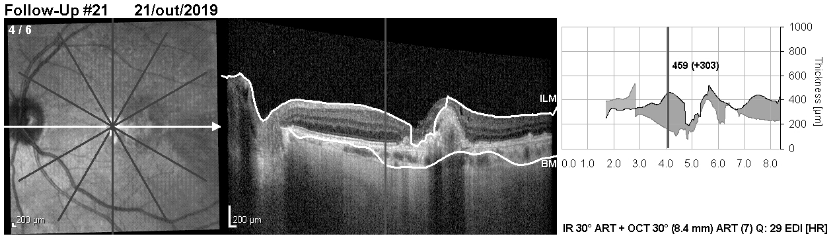

Follow-Up #22 03/dez/2019

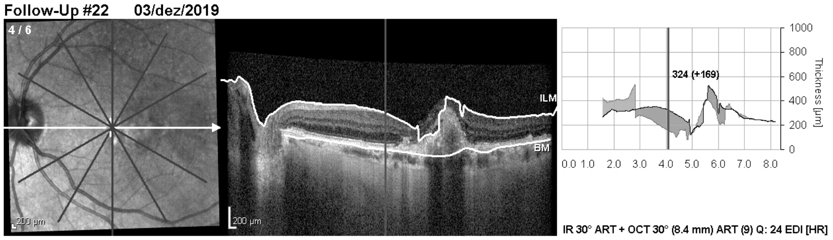

Follow-Up #23 05/mar/2020

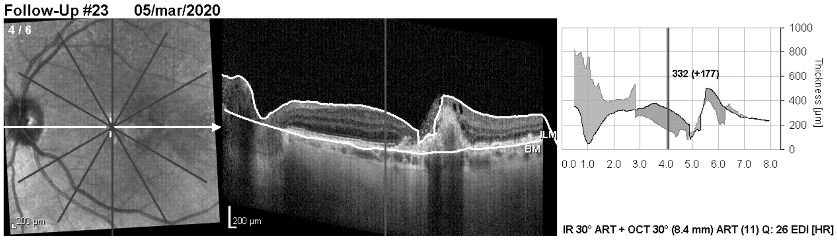

Notes:

Date: 03/01/2024 Signature:

Software Version: 6.12.4 *www.HeidelbergEngineering.com* *Retina Change Report, All Follow-Ups, Page 6/8*

Follow-Up #24 **27/abr/2020**

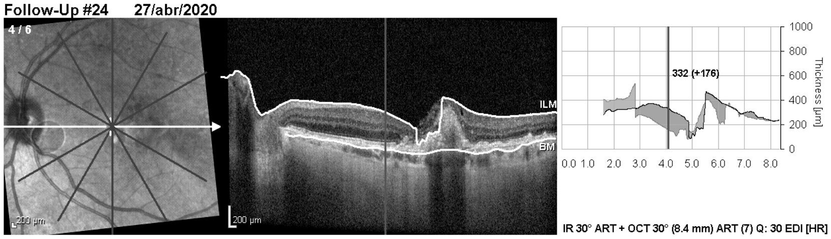

IR 30° ART + OCT 30° (8.4 mm) ART (7) Q: 30 EDI [HR]

Follow-Up #25 **13/jul/2020**

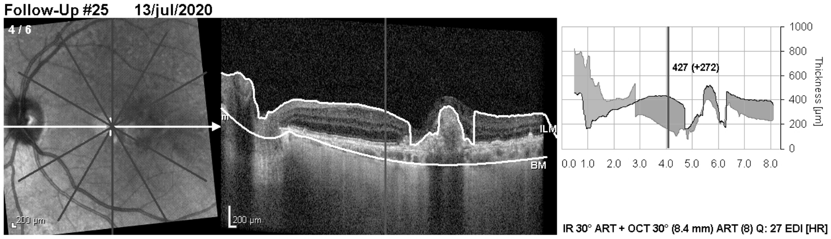

IR 30° ART + OCT 30° (8.4 mm) ART (8) Q: 27 EDI [HR]

Follow-Up #26 **26/ago/2020**

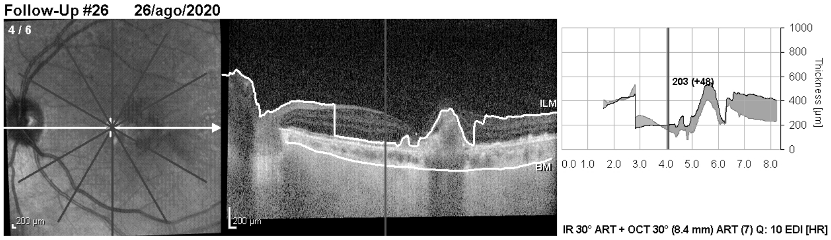

IR 30° ART + OCT 30° (8.4 mm) ART (7) Q: 10 EDI [HR]

Follow-Up #27 **08/out/2020**

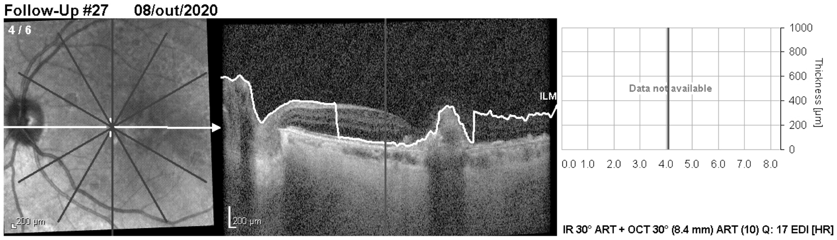

IR 30° ART + OCT 30° (8.4 mm) ART (10) Q: 17 EDI [HR]

Notes:	
Date: 03/01/2024	Signature:

Follow-Up #28 **29/jan/2021**

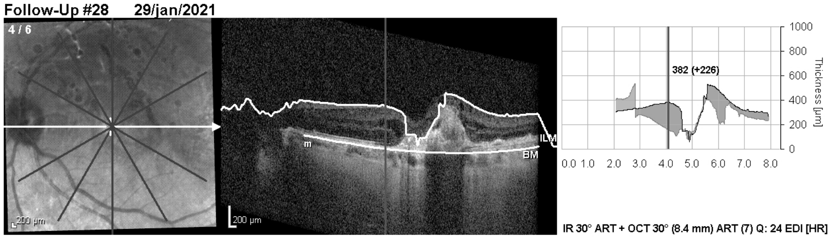

IR 30° ART + OCT 30° (8.4 mm) ART (7) Q: 24 EDI [HR]

Follow-Up #29 **01/abr/2021**

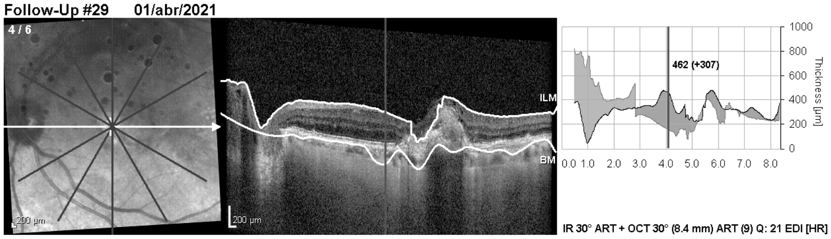

IR 30° ART + OCT 30° (8.4 mm) ART (9) Q: 21 EDI [HR]

Follow-Up #30 **20/mai/2021**

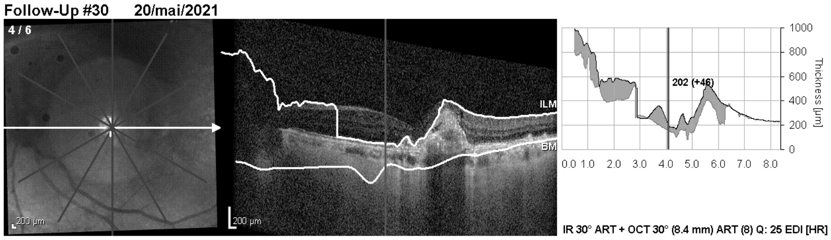

IR 30° ART + OCT 30° (8.4 mm) ART (8) Q: 25 EDI [HR]

Reference **24/mai/2021**

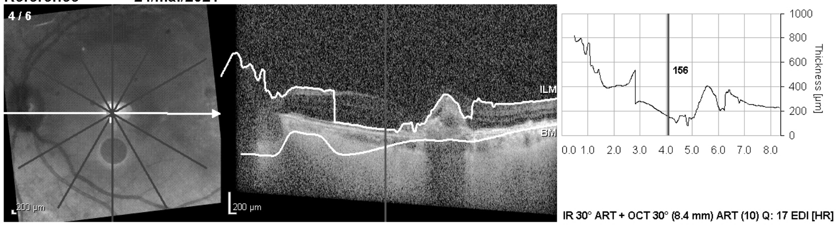

IR 30° ART + OCT 30° (8.4 mm) ART (10) Q: 17 EDI [HR]

Notes:

Date: 03/01/2024 Signature:

Software Version: 6.12.4 *www.HeidelbergEngineering.com* *Retina Change Report, All Follow-Ups, Page 8/8*

Baseline **13/jan/2022**

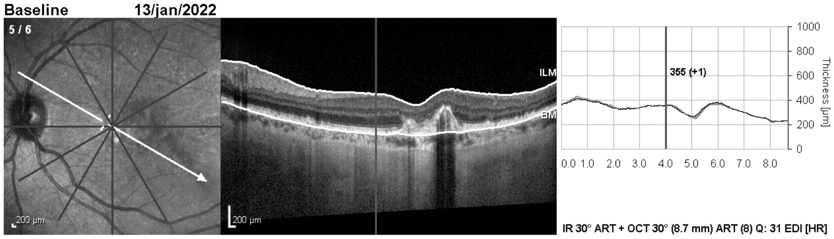

IR 30° ART + OCT 30° (8.7 mm) ART (8) Q: 31 EDI [HR]

Follow-Up #1 **02/mai/2022**

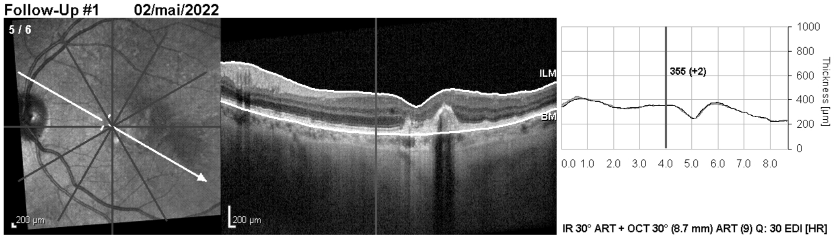

IR 30° ART + OCT 30° (8.7 mm) ART (9) Q: 30 EDI [HR]

Follow-Up #2 **01/jul/2022**

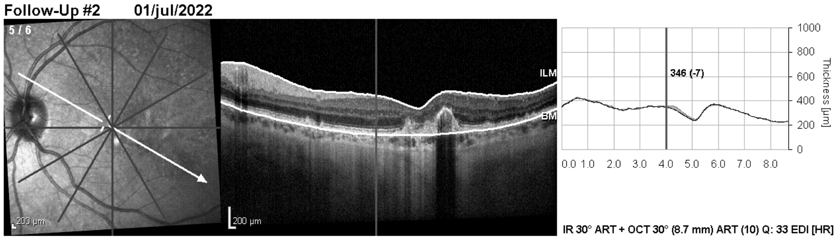

IR 30° ART + OCT 30° (8.7 mm) ART (10) Q: 33 EDI [HR]

Follow-Up #3 **26/ago/2022**

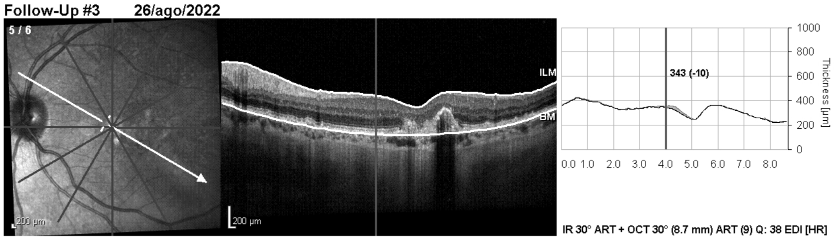

IR 30° ART + OCT 30° (8.7 mm) ART (9) Q: 38 EDI [HR]

Notes:

Date: 03/01/2024 Signature:

Software Version: 6.12.4 *www.HeidelbergEngineering.com* *Retina Change Report, All Follow-Ups, Page 1/3*

[illegible]

Follow-Up #4 **12/set/2022**

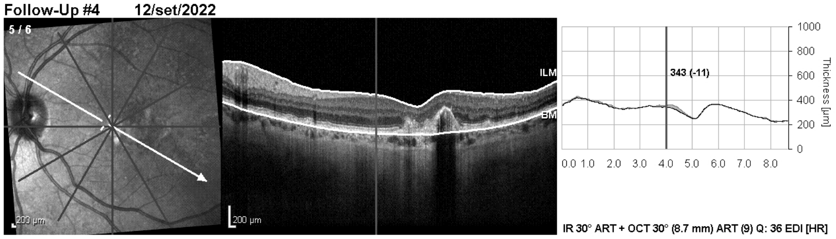

IR 30° ART + OCT 30° (8.7 mm) ART (9) Q: 36 EDI [HR]

Follow-Up #5 **24/nov/2022**

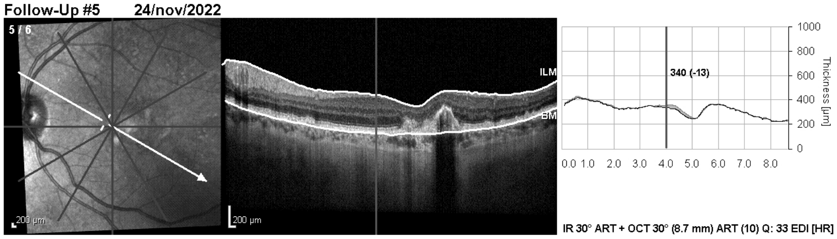

IR 30° ART + OCT 30° (8.7 mm) ART (10) Q: 33 EDI [HR]

Follow-Up #6 **27/fev/2023**

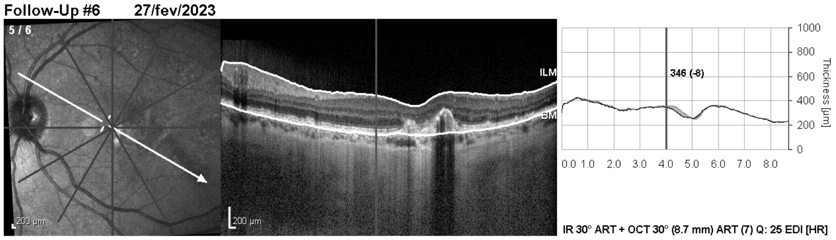

IR 30° ART + OCT 30° (8.7 mm) ART (7) Q: 25 EDI [HR]

Follow-Up #7 **21/abr/2023**

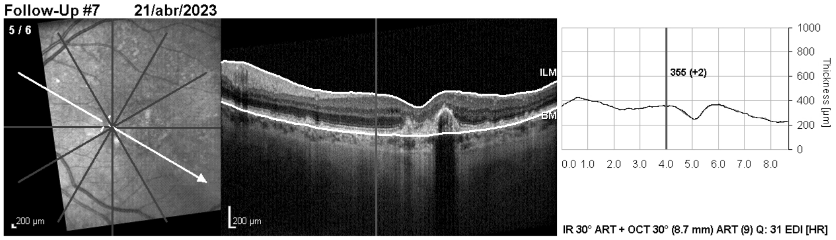

IR 30° ART + OCT 30° (8.7 mm) ART (9) Q: 31 EDI [HR]

Notes:	
Date: 03/01/2024	Signature:

Follow-Up #8 **19/jun/2023**

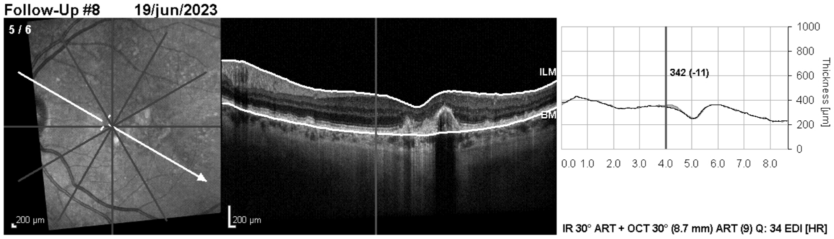

IR 30° ART + OCT 30° (8.7 mm) ART (9) Q: 34 EDI [HR]

Reference **06/out/2023**

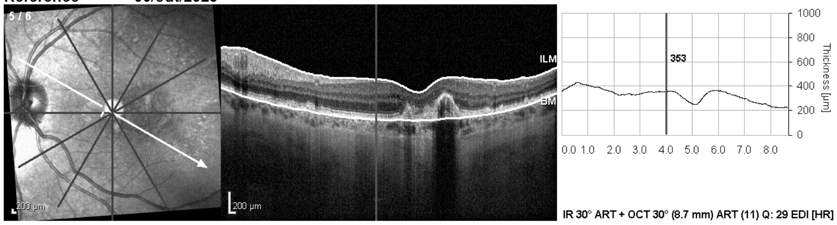

IR 30° ART + OCT 30° (8.7 mm) ART (11) Q: 29 EDI [HR]

Follow-Up #10 **27/nov/2023**

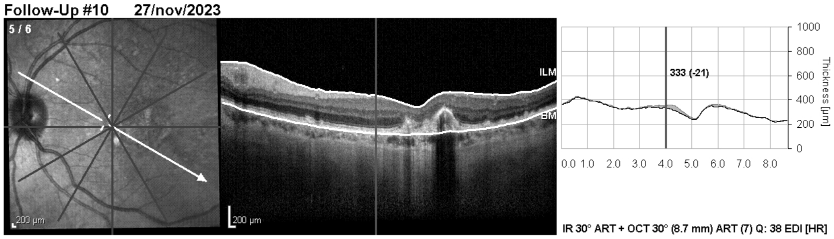

IR 30° ART + OCT 30° (8.7 mm) ART (7) Q: 38 EDI [HR]

Notes:

Date: 03/01/2024 Signature:
